# Remotely Controlled Surface Charge Modulation of Magnetoelectric Nanogenerators for Swift and Efficient Drug Delivery

**DOI:** 10.1021/acsomega.4c03825

**Published:** 2024-06-15

**Authors:** Nandan Murali, Simran Kaur Rainu, Arti Sharma, Soumik Siddhanta, Neetu Singh, Soutik Betal

**Affiliations:** † Department of Electrical Engineering, Indian Institute of Technology Delhi, Hauz Khas, New Delhi 110016, India; ‡ Center for Biomedical Engineering, Indian Institute of Technology Delhi, Hauz Khas, New Delhi110016, India; § Department of Chemistry, Indian Institute of Technology Delhi, Hauz Khas, New Delhi110016, India

## Abstract

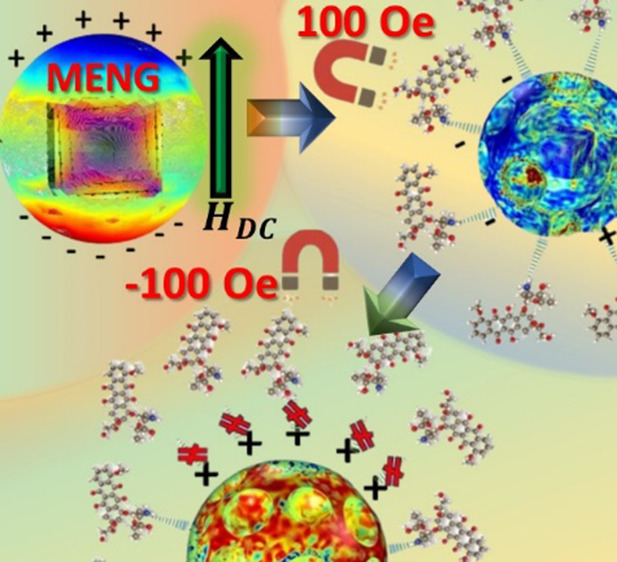

We have developed a highly efficient technique of magnetically controlled swift loading and release of doxorubicin (DOX) drug using a magnetoelectric nanogenerator (MENG). Core–shell nanostructured MENG with a magnetostrictive core and piezoelectric shell act as field-responsive nanocarriers and possess the capability of field-triggered drug release in a cancerous environment. MENGs generate a surface electric dipole when subjected to a magnetic field due to the strain-mediated magnetoelectric effect. The capability of directional magnetic field-assisted modulation of the surface electrical dipole of MENG provides a mechanism to create/break ionic bonds with DOX molecules, which facilitates efficient drug attachment and on-demand swift detachment of the drug at a targeted site. The magnetic field-assisted drug-loading mechanism was minutely analyzed using spectrophotometry and Raman spectroscopy. The detailed time-dependent analysis of controlled drug release by the MENG under unidirectional and rotating magnetic field excitation was conducted using field-emission scanning electron microscopy, energy-dispersive X-ray, and atomic force microscopic measurements. In vitro, experiments validate the cytocompatibility and magnetically assisted on-demand and swift DOX drug delivery by the MENG near MCF-7 breast cancer cells, which results in a significant enhancement of cancer cell killing efficiency. A state-of-the-art experiment was performed to visualize the nanoscale magnetoelectric effect of MENG using off-axis electron holography under Lorentz conditions.

## 1 Introduction

Cancer remains one of the most forbidding challenges in the medical field, demanding innovative approaches to combat persistent progression. The most common therapeutic intervention for cancer is chemotherapy. Indeed, a major drawback of chemotherapy is its lack of selectivity in targeting cancer cells, leading to systemic toxicity, the development of drug-resistant tumors, and insufficient drug delivery to tumor sites.^1^ This indiscriminate action of chemotherapy drugs affects both cancerous and healthy cells, resulting in various side effects and limiting the effectiveness of treatment.^2,3^ Consequently, there is a critical need for more targeted approaches to deliver chemotherapy drugs specifically to cancer cells while sparing healthy tissues, thereby minimizing toxicity and improving therapeutic outcomes. In past decades, the convergence of biomedical engineering and nanotechnology has signaled a new era for promising precision delivery systems for targeted therapeutics capable of revolutionizing cancer treatment.^4^ Formulating drug molecules within nanoscale carriers offers a compelling strategy to enhance the therapeutic potential of anticancer pharmaceuticals and overcome the chemotherapeutic side effects.^5−7^ Several active drug delivery systems are available, such as polymeric micelles,^8^ liposomes,^9^ lipoprotein-based carriers,^10^ nanoparticles,^11^ and dendrimers.^12^ These nanoparticle-based drug carriers exhibit notable advantages such as extended shelf life, leading to increased stability, and can facilitate a wide range of drug molecules within the particle matrix.^13,14^ Furthermore, these can accommodate both hydrophilic and hydrophobic substances, and nanoparticles allow for versatile routes of administration, encompassing oral intake and inhalation.^15−17^

Researchers have proposed a wide range of applications of drug-loaded nanoparticles designed to precisely target tumor sites.^18−20^ For instance, glutathione-responsive polyurethane nanoparticles loaded with the DOX drug, developed by Iyer et al., effectively treated xenograft A549 lung tumor models in mice.^21^ Additionally, Yang et al. recently demonstrated the effectiveness of tumor-specific PEGylated bilirubin nanoparticles loaded with drug dimers, which suppressed robust 4T1 breast cancer cells.^22^ Further, these drug nanocarriers can be classified based on the different triggering mechanisms such as magnetic-responsive,^23−25^ pH-responsive,^26^ light-responsive,^27^ enzyme-responsive,^28^ and hypoxia-responsive.^29^ Xu et al.,^30^ encapsulated DOX in poly(acrylic acid) stabilized with amorphous calcium carbonate nanoparticles for sustainable release of drug by sensing the acidic pHs environment zebrafish model for eradication of human hepatocarcinoma cell line (SMCC-7721). However, these pH-sensitive drug release systems do not provide precise control over drug release rates and can lead to unpredictable release kinetics.^31^ Additionally, the reliance on pH as a triggering mechanism may limit the applicability of these systems in certain physiological conditions in which pH fluctuations are minimal.^32^ Mahlert et al.^33^ investigated photodynamic therapy for treating gastrointestinal tumors. They achieved in vitro drug release by exposing nanoparticles containing a light-responsive polycarbonate and poly(lactic-*co*-glycolic-acid) loaded with the m-hydroxyphenyl photosensitizer to 365 nm UV light for 5 min at an energy level of 174 J cm^–2^. This resulted in a significant burst release of the drug in human colon cancer cell lines (HT-29-MTX). While the photothermal technique for eliminating tumor cells proves to be highly efficient, the complexity of laser irradiation in in vivo applications is highly impractical.^34,35^ T. Anajafi et al. engineered redox-sensitive polymersomes for targeted curcumin and DOX delivery to the nucleus in pancreatic cancer, enhancing drug accumulation at tumor sites.^36^ Employing a matrix metalloproteinase-7 (MMP-7) peptide linker, the researchers have activated nuclear localization signal (NLS) peptides selectively in high MMP-7 concentrations within tumors. The nanoparticulate system demonstrated heightened toxicity toward human pancreatic cancer cell lines (BxPC-3 and AsPC-1), compared with normal cells, despite nonselective NLS peptides. However, reactive oxygen species potentially cause oxidative stress and damage to cells and tissues, leading to side effects in the human body,^37^ and also different redox potentials in various organs lead to premature drug release.^38^ Park et al.^39^ proposed an acoustically mediated porous microrobot encapsulated with Fe_3_O_4_ nanoparticles conjugated with a 5-fluorouracil anticancer drug. These microrobots were brought to the targeted area by applying 20 mT and 7 Hz via corkscrew locomotion. The ultrasound beam of the 500 kHz sinusoidal wave at 1 W cm^–2^ was used, and the maximum drug release was observed in burst and constant mode. Despite numerous proposed advanced nanocarrier-based drug delivery systems, there are limitations in the controllability and speed of drug release at the targeted site, the ease of applying an external stimulating field for field-triggered drug release, and the maneuverability of the drug-loaded nanocarrier to the targeted site.^40,41^ Furthermore, the initial use of magnetic NPs as drug carriers had drawbacks, such as the generation of free radicals with iron oxide and adverse effects on cells in vitro from high magnetic response leading to the production of metallic ions.^42^

Rincón-Iglesias et al. fabricated Fe_3_O_4_@Au core–shell nanorods with a 5 nm shell thickness for applications in magneto- and photothermia.^43^ Taheri-Ledari et al. proposed an octahedral morphology with a core–shell structure made of Fe_3_O_4_/BioMOF-13 for delivering doxorubicin in breast cancer treatment.^44^ However, the use of spherical core–shell structures like MENG offers several advantages, including better conjugation with drugs, receptors, and ligands, increased chemical and thermal stability, and lower cytotoxicity.^45,46^

Recently, magnetoelectric nanoparticles have emerged as front-runners in the pursuit of high-specificity, field-controlled drug delivery to cancer cells under the guidance of a magnetic field.^47−49^ Betal et al. have experimentally verified the magnetically assisted nanorobotic functionalities of magnetoelectric nanoparticles in a biocellular environment, which includes targeted single-cell electroporation,^50^ cell transport to targeted location, and precise cell targeting.^51^ In another extensive study, Betal et al.^52^ have demonstrated the nanovehicle functionality of magnetoelectric nanoparticles for accurate cell targeting. The high-speed propulsion of magnetoelectric nanovehicles was actuated by the magnetic field and biocellular electric field sensing in the human epithelial cell environment. By utilizing the unique properties of magnetoelectric (ME) materials, these nanoparticles can respond to external magnetic fields with exceptional sensitivity and the capacity to transduce this stimulus into controlled drug release.^53,54^ This dynamic ability holds the potential to significantly improve the efficacy of cancer therapies while minimizing off-target effects, thus addressing critical challenges faced by conventional drug delivery methods such as low-pass effect, premature drug release at nontargeted sites, and low bioavailability of therapeutic agents.^55^ The effectiveness of the magnetoelectric material can be evaluated by investigating its ME coefficient (α). In a heterostructure, this coefficient is greatly influenced by the mechanical interaction between the ferromagnetic and ferroelectric phases, as well as the shape, size, and dimensions of the composite material.^56,57^ At the nanoscale, the ideal shape for achieving the highest level of mechanical interaction between these two phases is a core–shell-like heterostructure. In this configuration, the ferromagnetic core is enclosed by a ferroelectric material shell, offering numerous advantages, and producing favorable outcomes compared with other nanostructures.^51^

**Figure 1:**
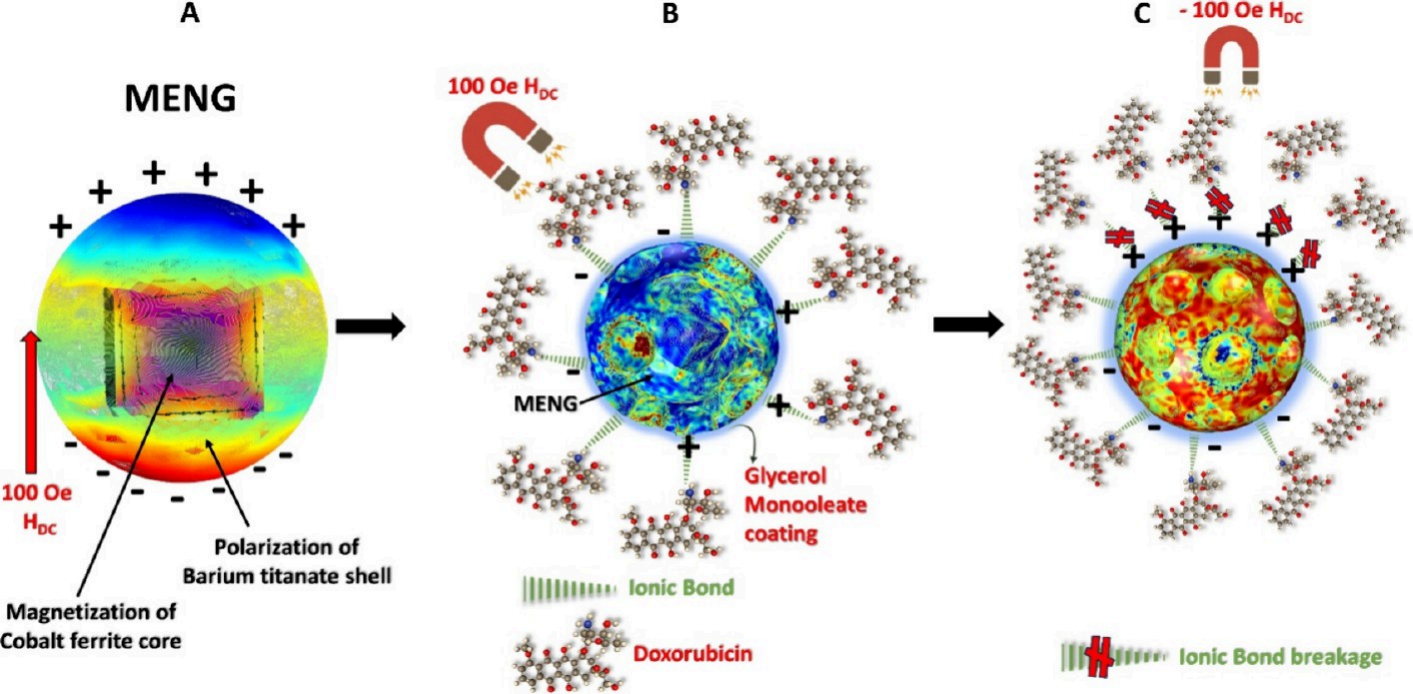
Schematic representation of the controllability of drug loading and release using a MENG. (A) Electric dipole created on the surface of MENG due to ME effect excited with *H*_DC_ = 100 Oe. (B) Ionic bond formation of the DOX drug molecule on the surface of MENG under the influence of 100 Oe magnetic field applied. (C) Directional ionic bond breaking of the DOX drug molecule on the surface of MENG due to directional magnetic field application with reverse polarity (*H*_DC_ = −100 Oe).

This research demonstrates a highly efficient and swift on-demand technique for controlled drug loading and targeted release of DOX using a weak direct current magnetic field (*H*_DC_). This study explores the potential of a magnetoelectric nanogenerator (MENG), which is a core–shell heteronanostructure and comprises a crystalline CoFe_2_O_4_ (CFO) core coated with a crystalline BaTiO_3_ (BTO) shell, as a nanocarrier for the anticancer drug DOX and assesses their performance in vitro when subjected to an external magnetic field. When subjected to a +100 Oe magnetic field, the MENG generates an electric dipole on the surface due to its unique ME behavior. This arises from the deformation experienced by the magnetostrictive CFO core under H_DC_ excitation, which induces compressive stress on the piezoelectric BTO shell. This stress leads to the generation of electric dipoles on the surface of MENG with polarity and amplitude directly dependent on the polarity, amplitude, and direction of the applied *H*_DC_, as shown by the schematical representation in Figure 1A. The total electric polar charge (δ_s_) generated on the surface of MENG can be quantified mathematically as, , where α is the magnetoelectric coefficient of the MENG, ε_o_ is the permittivity of vacuum, ε_r_ is the permittivity of the material of the shell of the core–shell nanostructured MENG, d is the diameter of the MENG, and *H*_DC_ is the intensity of the applied DC magnetic field. Betal et al. have presented the electric dipole created under magnetic field excitation on the surface of a single core–shell magnetoelectric nanostructure by off-axis electron holography measurements.^50^ Moreover, under oscillating magnetic field excitation, the core vibrates at the same frequency as the oscillating magnetic field, resulting in the generation of an oscillating dipole on the surface of the core–shell magnetoelectric nanostructure. Hence, the oscillating electric field generated by this core–shell magnetoelectric nanostructure has the same frequency as the applied oscillating magnetic field.^51,52^

With the application of magnetic field *H*_DC_ = 100 Oe directed in a specific orientation, such as clockwise, an ionic bond is formed between the MENG and the ions in the DOX drug molecule (typically interconnected in nanochains) as presented in the schematical representation in Figure 1B. Additionally, glyceryl monooleate (GMO) coating acts as a stabilizing agent to firmly hold the loaded drug molecule after the removal of the magnetic field. To achieve drug release, symmetry dictates that the ionic bond should be disrupted by replicating the field sequence in the opposite direction. However, this simplified scenario does not account for the variability in the directions of the nanoformulations. Ideally, by employing a rotating magnetic field with opposite polarity (i.e., *H*_DC_ = −100 Oe), the magnetostrictive core exerts expansive stress on the shell that uniformly reverses the electric dipole direction on the surface of the MENG and results in a more consistent and effective ionic bond-breaking across the surface of the nanocarriers and the drug as shown in Figure 1C, thereby enhancing efficiency, controllability as well as swiftness of the drug release. This modulation capability of the surface electric dipole with varying polarity and direction of applied magnetic field leads to on-demand and swift ionic bond creation and disruption with ions of the DOX molecule and leads to rapid loading and release of DOX. These DOX-loaded nanocarriers, when subjected to a low-intensity DC magnetic field, effectively release the anticancer drug and thus kill cancerous cells. Figure 1 provides a detailed schematic representation of the concept of on-demand drug loading and release under the influence of *H*_DC_.

## 2 Experimental Section

### 2.1 Materials

Cobalt ferrite (CoFe_2_O_4_), titanium (IV) isopropoxide (with a purity of 99.99%), barium carbonate (BaCO_3_, with a purity of 99.99%), citric acid (99.99% pure), GMO, and doxorubicin hydrochloride (DOX) were commercially procured from Sigma-Aldrich. Dulbecco’s modified Eagle’s medium (DMEM), antibiotic–antimycotic solution, fetal bovine serum (FBS), Dulbecco’s phosphate-buffered saline (DPBS), calcein-AM, propidium iodide (PI), 3-(4,5-dimethylthiazol-2-yl)-2,5-diphenyltetrazolium bromide (MTT), and the Pierce BCA Protein Assay Kit were procured from Thermo Fisher Scientific. The NIH-3T3 and MCF-7 cell lines were obtained from NCCS, Pune (India). All chemicals were of analytical grade and used in their as-received state. This study maintained a standard of using high-purity reagents and commercially available cell lines to ensure the reliability and reproducibility of experimental results.

### 2.2 Fabrication of Core–Shell MENG

The MENGs were synthesized via the temperature-controlled Pechini method.^45,46^ A solution of barium citrate was prepared by mixing 30 mL of ethanol, 0.058 g of BaCO_3_, and 0.2 g of citric acid and stirred for 2 h, while a solution of titanium citrate was obtained by mixing 30 mL of ethanol, 0.096 mL of titanium isopropoxide, and 2 g of citric acid and stirred for 2 h. These solutions were then mixed and thoroughly stirred for 2 h at a constant 40 °C using a magnetic stirrer. Subsequently, 0.2 g of commercially sourced CFO nanoparticles was sonicated for 2 h in 40 mL of ethanol to prevent agglomeration. Further, both solutions were mixed, and this well-dispersed mixture was dried at 80 °C overnight while stirring continuously using a mechanical stirrer. The resulting particles were grinded finely by using a mortar and then calcinated at 780 °C for 6 h in a temperature-controlled furnace. By maintaining a cooling rate of 6 °C per minute, the nanoparticles were gradually brought down from 780 °C to RT, leading to the growth of the BTO shell on the CFO surface resulting in the formation of MENG.

### 2.3 Preparation of GMO-Coated MENG (GMO@MENG)

To facilitate and stabilize the loading of the DOX drug, the surface of MENGs was coated with GMO. This process involved adding 1 mg of GMO to 5 mg of MENGs in 5 mL of DPBS. The mixture was subsequently incubated for 12 h with slow rotation to ensure an even coating. After incubation, the MENGs were subjected to centrifugation for 20 min at 10 °C (rpm = 10,000). The resulting pellet was washed three times with an ethyl acetate and acetone (70:30) solution in the ratio of 70:30 to thoroughly eliminate any extra, unbound GMO. Finally, the resulting pellet was subjected to freeze-drying for 48 h and stored for future use.

### 2.4 Spectrophotometry Analysis of DOX Loading on the Surface of GMO-Coated MENG (DOX-GMO@MENG) and Magnetic Field Exposure

Following the addition of 5 μL of DOX drug (2 mg/mL) to 1 mL of GMO@MENG solution (0.5 mg/mL) in DPBS buffer, the solution was incubated for 3 h with gentle stirring and exposed to the clockwise rotational magnetic field (*H*_DC_ = 100 Oe) using an electromagnet for 5 min. Subsequently, the solution underwent centrifugation at 10,000 rpm for a time interval of 10 min at 10 °C to eliminate any unbound drug. The resulting supernatant was separated, and absorbance was recorded spectrophotometrically at 480 nm using a microplate reader (Biotek Synergy H1Multimode plate reader). Moreover, the pellet containing DOX-GMO@MENG-H was washed three times with DPBS and freeze-dried for further studies. The DOX loading percentage was recorded for both magnetically exposed and unexposed GMO@MENG samples named DOX-GMO@MENG-H and DOX-GMO@MENG respectively. The drug-loading efficiency was calculated by the following equation:
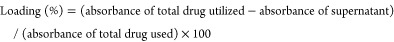


### 2.5 Sample Preparation for Raman Spectroscopy Measurements

The preparation for Raman spectroscopy involved drop-casting samples on an aluminum-wrapped glass slide. The samples include DOX, GMO@MENG, and a solution containing 5 μL of DOX drug into 1 mL of GMO@MENG solution and incubated for 3 h. Subsequently, GMO@MENG-DOX was subjected to the magnetic field of 100 Oe in the clockwise direction for 5 min. Following this, Raman measurements were carried out using an Olympus Horiba XploRa Raman spectrometer equipped with a 785 nm laser and a 50× infinity-corrected objective lens featuring a numerical aperture (NA) of 0.50. The accumulation time was set at 10 s, and the laser power was maintained at 10 mW. After data acquisition, postprocessing and spectral smoothing were executed using the Savitzky-Golay method with 5 adjacent points, facilitated by Origin 2020b software. Principal component analysis (PCA) was carried out for further analysis using MATLAB software. This comprehensive approach ensured precise and detailed insights into the Raman spectra, contributing to the overall characterization of the synthesized materials.

### 2.6 Sample Preparation for HRTEM and Off-Axis Electron Holography

The preparation of samples for HRTEM involved dissolving 1 mg of MENGs in 10 mL of ethanol. Subsequently, this solution underwent an hour-long sonication to prevent any agglomeration, followed by 5 min of vortexing. Afterward, 20 μL of the solution was carefully pipetted out and dispensed as a single droplet onto a carbon-coated copper grid. This grid was air-dried overnight in preparation for imaging. The microstructure images of MENG were captured using the JEM-ARM200F NEOARM Atomic Resolution Analytical Electron Microscope, and a detailed discussion has been presented in Section 3.1. A similar grid preparation was undertaken for off-axis electron holography measurements, and a thorough discussion of the results can be found in the results and discussion. This meticulous sample preparation process ensured the acquisition of nonagglomerated high-quality images and accurate data for the subsequent analysis and discussion.

### 2.7 Method for Magnetic and Electrostatic Phase Extraction through Off-Axis Electron Holography under Lorentz Field Conditions

Off-axis electron holography is performed using scanning transmission electron microscopy (STEM) with a probe-corrected microscope (JEOL-ARM200F). The STEM was equipped with a biprism (thin platinum wire) connected to a voltage source, a Lorentz lens attached to the microscope, and Gatan Digital micrograph software with a beta version of HoloWorks 5.0.7. With respect to magnetic materials, electron holography necessitates to be performed using Lorentz mode under field-free conditions, which involves turning off the objective lens to preserve the undisturbed remnant magnetization state of the sample by employing the Lorentz lens and subject to a magnetic field of 1.5 T. The residual magnetic field of the objective lens was measured to be approximately 50 Oe. The holograms must be captured using high fringe contrast (34%), which can be varied with an application of bias voltage to the biprism. Specifically for MENG, a biprism voltage of 40 V was used to cover the object’s field of view. The Fresnel fringes created had a width of 140 nm. In our experiment, the orientation of a single MENG aligns longitudinally parallel to the interference fringes. To enhance the phase resolution, single exposures with exposure times ranging from 2 to 4 s were used for registered hologram acquisition.

The holograms were captured using Gatan’s Digital Micrograph software and reconstructed using a beta version of HoloWorks 5.0.7, featuring a function for extracting magnetization and electrostatic potential from phase images at several frames per second. The numerical reconstruction of each retrieved phase involved a reference hologram and an object hologram to eliminate the impact of the perturbed reference wave. To achieve the electromagnetic phase separation between the magnetization and the electrostatic potential, manual flipping of the sample was done (up and down) by flipping the TEM grid containing the MENG samples. The phase extracted from the up or down flip state of the sample contains both magnetic and electrostatic phases. The electrostatic potential generated by a single MENG was extracted by adding the phase maps obtained from the two flip states (up and down). Detailed discussions regarding this process can be found in Section 3.1 and Figure S1 of the Supporting Information.

### 2.8 Sample Preparation for FESEM Measurements

A solution of DOX-GMO@MENG-H and DPBS buffer was prepared and divided into 4 batches containing 2 mL each. Batch 1 was kept untouched, Batch 2 was exposed to 15 s of unidirectional magnetic field (*H*_DC_ = −100 Oe), Batch 3 was exposed to 45 s of unidirectional magnetic field (*H*_DC_ = −100 Oe), and Batch 4 was exposed to a rotating magnetic field (*H*_DC_ = −100 Oe) for 2 min. 5 μL of solution for each batch, i.e., batches 1–4, was drop-cast on a Si wafer and dried overnight. 5 μL of solution for each batch, i.e., batches 1–4, was drop-cast on a Si wafer and dried overnight. DOX-GMO@MENG-H coated on a Si substrate was sputtered with 3 nm of platinum for enhanced image contrast without affecting the sample appearance. Microscopic and energy-dispersive X-ray spectroscopy (EDS) analyses were conducted using the JSM-7800F Prime field-emission scanning electron microscope (FESEM). A detailed discussion of the findings from these analyses can be found in Section 3.3 of the study, providing insights into the morphological and elemental characteristics of the DOX-GMO@MENG-H samples under different magnetic field exposures.

### 2.9 Atomic Force Microscopy Measurements

Initially, (1 cm × 1 cm) Si wafer was subjected to plasma cleaning for organic, C, and O impurities removal. Subsequently, a solution of DOX-GMO@MENG-H and DPBS buffer was prepared and divided into 2 batches containing 2 mL each. Batch 1 was exposed to 45 s of unidirectional magnetic field (*H*_DC_ = −100 Oe), and Batch 2 was exposed to a rotating magnetic field (*H*_DC_ = −100 Oe) for 2 min. 5 μL of solution for each batch, i.e., batches 1 and 2, was drop-cast on a Si wafer and dried overnight. Next, by using the Asylum Research MFP3D-BIO-AFM system, a tapping mode scan was conducted on the 3 μm × 3 μm area of the specimen to acquire both topographic and phase information. The low moment AFM tip utilized was constructed from standard pyramidal-shaped etched silicon, with dimensions of 225 × 30 × 3 μm (*L* × *W* × *t*). The tip’s resonant frequency was determined to be 340 kHz, and its radius measured 40 nm (Bruker). Detailed findings from the AFM analysis are discussed in the Result and Discussion, providing insights into the surface characteristics and morphology of the specimens under different magnetic field exposures.

### 2.10 Spectrophotometry Analysis of Drug Release

The study of drug release involved the exposure of unidirectional or rotating DC magnetic fields over distinct time intervals. DOX-GMO@MENG-H samples were utilized in all experiments carefully after filtering out any unbound drug. DOX-GMO@MENG-H samples were dispersed in DPBS at a concentration of 1 mg/mL and subjected to an external magnetic field (*H*_DC_ = −100 Oe) at room temperature for 5 min. Subsequently, these samples were then centrifuged for 5 min at low rpm, and the resulting supernatants were subjected to spectrophotometric analysis. The absorbance values corresponding to the amount of drug released into the supernatant, influenced by the presence and absence of magnetic stimuli, were recorded at 480 nm. The data were used to plot a bar graph representing the relative amount of drugs released. This analytical approach allowed for a quantitative assessment of the drug release behavior under the influence of magnetic stimuli and provided valuable insights into the controlled release capabilities of the DOX-GMO@MENG-H samples.

### 2.11 Cell Culture

The human breast cancer cell line (MCF-7) and murine fibroblast cell line (NIH-3T3) were cultured in DMEM. The medium was supplemented with 1% antibiotic-antimycotic solution and 10% FBS. The cell cultures were maintained in a CO_2_ incubator at 37 °C. This standard culture condition provided the necessary nutrients and environment for the growth and propagation of the cell lines, ensuring their viability and allowing for experimental studies in a controlled and supportive environment.

### 2.12 In Vitro Cytocompatibility Studies

The cytocompatibility of MENGs and GMO@MENGs was evaluated in NIH-3T3 cell line via MTT and live–dead assay. 5000 cells/well were seeded in a 96-well plate, and cells were allowed to grow at 37 °C. After 24 h of incubation, the MENGs were added at concentrations varying from 50 to 200 μg/mL to each well. The cell viability in the presence of these MENGs was evaluated after a 24 h incubation period and untreated cells were kept as control. The assay was performed in triplicates. The cellular metabolic activity was assessed using a colorimetric MTT assay. The cells were treated with MTT solution for 4 h and the obtained purple formazan crystals were dissolved in DMSO. The absorbance values at wavelength 570 nm were recorded using a microplate reader (Biotek Synergy H1Multimode plate reader). Live–dead assay was achieved by staining the MENGs treated cells with 2 μM Calcein-AM (stains live cells green) and 2 μM propidium iodide (stains dead cells red) dyes for 20 min and visualizing them under a fluorescence microscope (Carl Zeiss) to assess cell viability.

### 2.13 Protein Adsorption Assay Using Bovine Serum Albumin

Different concentrations (50, 70, and 100 μg/mL) of MENGs and GMO@MENGs were incubated in 1 mg/mL bovine serum albumin (BSA) solution at 37 °C. Postincubation for 0.5, 1, 6, and 24 h, the samples were centrifuged, and the supernatant was collected to analyze the amount of BSA present in it using the bicinchoninic assay (BCA) assay. The optical density (OD) at 562 nm was recorded using a microplate reader, and the percent BSA adsorbed was then calculated using the formula:
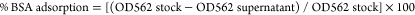


### 2.14 Anticancer Activity Monitoring of DOX@GMO-MENGs

Anticancer activity of DOX-GMO@MENG-H against the breast cancer cell line MCF-7 was assessed in the presence and absence of an external magnetic field. MCF-7 cells at a density of 5000 cells per well were seeded in a 96-well tissue culture plate and cultured for 24 h in complete media in a CO_2_ incubator at 37 °C. After 24 h, the culture media was substituted by media containing different concentrations (0, 100, and 200 μg/mL) of DOX@GMO-MENGs. Two such sets were prepared in triplicates; one was exposed to rotational DC magnetic field of −100 Oe external magnetic field for 2 min at 6 h of time intervals and the other set was not exposed to any external magnetic field. The following day, the anticancer effect of the doxorubicin released from the DOX-GMO@MENG-H in both the sets was evaluated by measuring the percentage of live cells using MTT assay and by visualizing live and dead cells (stained with Calcein-AM and PI dyes, respectively) under a fluorescence microscope.

### 2.15 Statistical Analysis

The results were presented as the mean ± standard deviation. A paired *t* test was performed to evaluate the difference between the two data sets. The difference between the data sets with a two-tailed *p*-value of less than 0.05 was considered to be statistically significant (*p < 0.05 was considered to be significant).

## 3 Results and Discussion

### 3.1 Microstructure and Nanoscale Magnetoelectric Property Analysis of MENG

**Figure 2:**
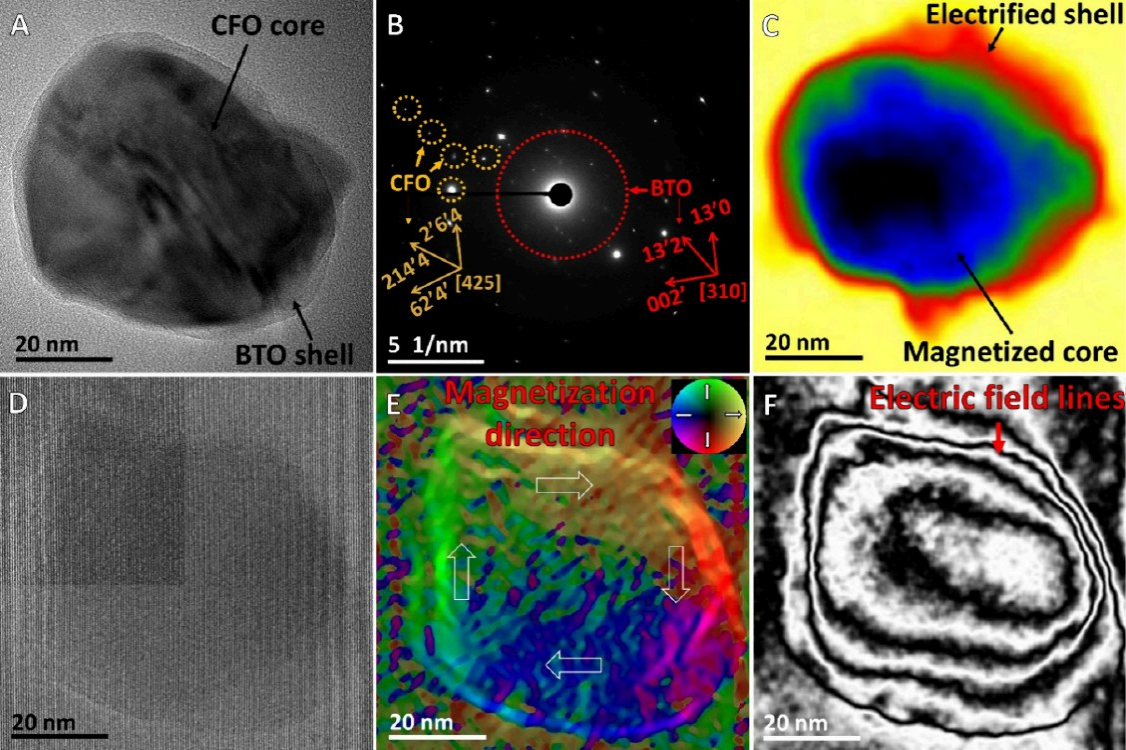
(A) HRTEM image of a single core–shell nanostructured MENG shows a distinct core coated with a thin shell. (B) Selected area electron diffraction shows a single crystal of CFO and BTO with lattice planes and zone axis mapped. (C) Electrification of shell and magnetization of the core when excited in situ with 1.5 T magnetic field of Lorentz lens and revealed using off-axis electron holography measurement. (D) Hologram attained from Off-axis electron holography measurements in the Lorentz field (1.5 T) condition containing MENG phase information in Fresnel fringes created by electron diffraction. (E) Magnetization direction of the MENG extracted from phase information in Figure 2D with direction indicational color map (inset). (F) Electric field lines on the surface of MENG generated due to the strain-mediated nanoscale magnetoelectric effect. Image extracted from phase information in Figure 2D.

The core–shell nanostructured MENG with a CFO core coated with a BTO shell was synthesized using Pechini’s method. The microstructure images of MENG were captured using high-resolution transmission electron microscopy (HRTEM) (JEM-ARM200F NEOARM atomic resolution analytical electron microscope). HRTEM image of a single MENG shows the presence of a core–shell structure (Figure 2A) and confirms a distinct contrast between the darker core and the lighter shell of the MENG. The core, with a diameter of ∼80 nm, is enveloped by a consistently uniform shell measuring ∼5 nm. The observed size of the MENG from the HRTEM analysis is approximately 90 nm. Furthermore, the average peak size distribution and zeta potential of MENG were determined to be approximately 90 nm and −15 mV, respectively, as observed through dynamic light scattering and zeta potential measurement (Figure S2 of the Supporting Information) and atomic force microscopy (Figure S3 of the Supporting Information). In Figure 2B, the selected area electron diffraction (SAED) patterns of the fabricated MENG are presented, which contains the crystalline lattices of both the BTO shell and the CFO core. The SAED pattern indexing was performed using JEMS software, revealing the zone axis as [4 2 5] and [3 1 0] for CFO and BTO, respectively. The lattice parameters indicate the spinel lattice of the CFO core and the tetragonal lattice of the BTO shell in the MENG. The crystallinity and the phase analysis using X-ray diffraction also reveal cubic-spinel and tetragonal phases of CFO and BTO, respectively (Figure S4 of the Supporting Information). The ferromagnetic properties of the CFO and MENG were analyzed using a vibrating sample magnetometer (VSM). The remnant magnetization (*M*_r_) and coercive field (*H*_C_) were determined to be 20.71 emu/g and 1756 Oe, whereas *M*_r_ and *H*_C_ for MENG were found to be 5.55 emu/g and 661 Oe, respectively (Figure S5 of the Supporting Information). The decreased value for the MENG observed compared with pure CFO can be attributed to the presence of nonmagnetic BTO shells that are incorporated into the CFO cores.^58^

Further off-axis electron holography (OEH) was performed using scanning transmission electron microscopy (STEM) with a probe-corrected microscope (JEOL-ARM200F). The STEM was equipped with a biprism (thin platinum wire) connected to a voltage source, a Lorentz lens attached to the electron microscope, and Gatan Digital micrograph software with a beta version of HoloWorks 5.0.7. Using the Lorentz lens, a single MENG was subjected in situ to a 1.5 T magnetic field, which is much higher than the observed saturation magnetic field measured using VSM measurements (Figure S5 of the Supporting Information). The OEH using STEM with attached biprism connected to a voltage supply was used to visualize the magnetization and nanoscale magnetoelectricity generated due to magnetic field exposure in a single MENG. The process of OEH under the Lorentz condition is described in detail in Section 2.7 as well as in Figure S1 of the Supporting Information. The phase extraction reveals the electromagnetic phase distinction of the core and shell of MENG due to the magnetization of the CFO core and the electrostatic potential created on the BTO shell, as presented in Figure 2C. The 1.5 T in situ magnetic field exposure by the Lorentz lens results in the shape deformation of the CFO core due to the magnetostriction property of the spinel CFO lattice. This exerts strain on the single crystalline perovskite lattice of the BTO shell. This strain results in the directional displacement of the titanium atom in each BTO lattice, creating an electrical dipole in each lattice because of the piezoelectric property of the BTO shell. Since BTO is in single crystalline form as observed in the selected area electron diffraction, all the electric dipoles created in each BTO lattice are unidirectional, resulting in the creation of a net electrostatic potential at the surface of the MENG. This strain-mediated magnetoelectricity generated in the core–shell nanostructure of MENG due to 1.5 T magnetic field excitation from the Lorentz lens was recorded using off-axis electron holography. The hologram presented in Figure 2D consists of the Fresnel fringes along with the electromagnetic phase information on a single MENG. The biprism with voltage supply attached to the STEM is used to generate the Fresnel fringes containing the electromagnetic phase information on MENG by electron diffraction of the transmitted electron wave from the MENG sample under observation. The electromagnetic phase was extracted from the recorded hologram with the technique described in detail in Section 2.7. To achieve the electromagnetic phase separation between the magnetization and the electrostatic potential, manual flipping of the sample was done (up and down) by flipping the TEM grid containing the MENG samples and analyzing using OEH. The phase extracted from the up or down flip state of the sample contains both magnetic and electrostatic phases. The electrostatic potential generated by a single MENG was extracted by adding the phase maps obtained from the two flip states (up and down). The holograms of the 2 flipped phases of the MENG were used to extract the magnetization direction (Figure 2E) and electric field lines generated on the surface of MENG (Figure 2F). The magnetization direction of a single MENG is highly dependent on the direction of the magnetic field due to the ferromagnetic property of the MENG. Hence the magnetostriction in the CFO core can be either compressive or expansive, depending on the magnetic field direction or polarity. Hence the stress exerted on the BTO shell by the CFO core will also be compressive or expansive and depends on the magnetic field direction. Hence the single crystalline BTO shell generates an electric dipole with the polarity direction strictly dependent on the direction or polarity of the applied magnetic field. Hence the OEH measurement proves the capability of magnetic field-assisted modulation of polarity of the electric dipole on the surface of a single MENG.

### 3.2 Raman Spectroscopic Analysis of Drug Conjugation on the MENG Surface

**Figure 3:**
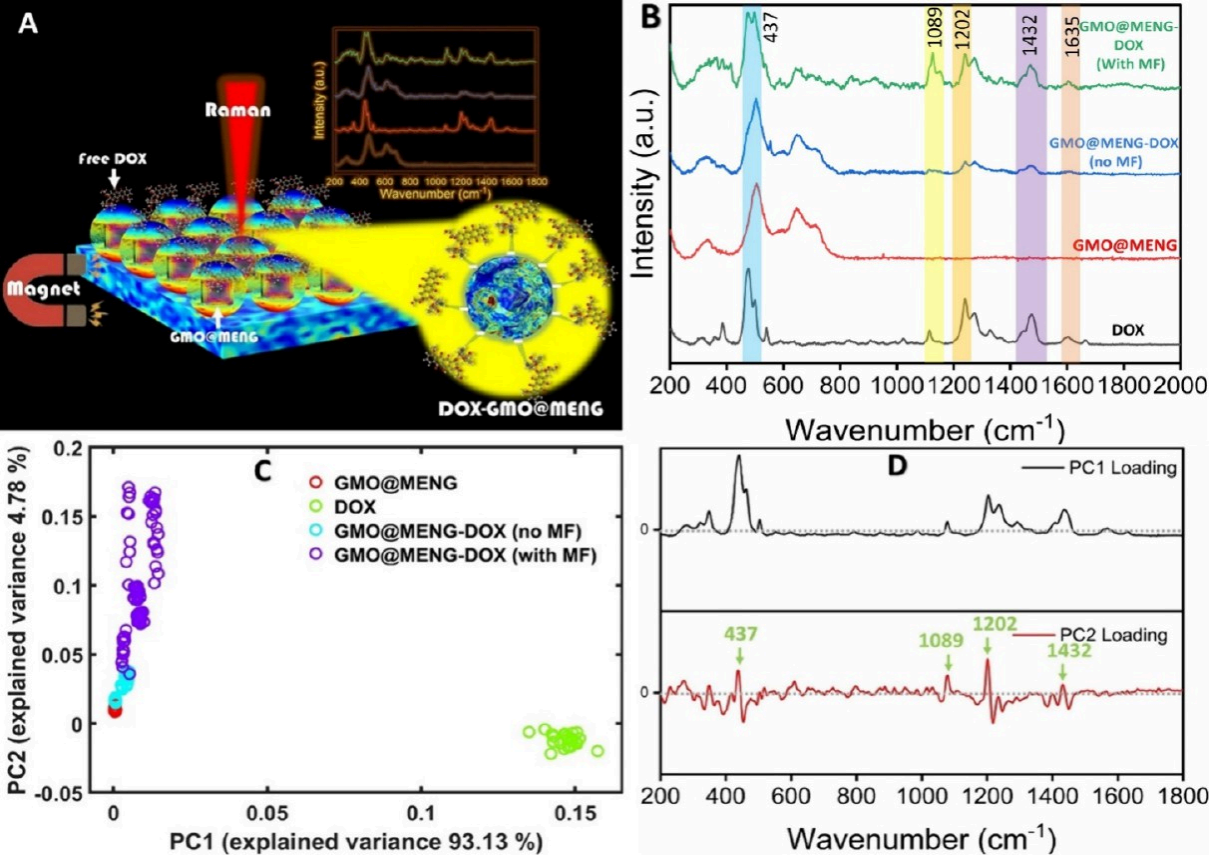
Raman spectroscopic measurements of DOX drug conjugation on GMO@MENG under the influence of *H*_DC_. (A) Schematic illustration of the application of +100 Oe magnetic field during Raman measurements to analyze the creation of ionic bonding of DOX molecule on the surface of GMO@MENG. The Raman spectra were acquired using a 785 nm laser excitation source, ensuring detailed molecular information, and facilitating the analysis of interactions between GMO@MENG and DOX, particularly in the presence of a magnetic field. (B) Raman spectra for DOX, GMO@MENG, GMO@MENG with DOX, and GMO@MENG with DOX in the magnetic field, represented in black, red, blue, green, and orange, respectively. The shaded portions in different colors indicate changes in peaks, which also correlate with the loading plots. (C) Principal component analysis (PCA) cluster plots for GMO@MENG, DOX, GMO@MENG with DOX, and GMO@MENG with DOX in the magnetic field are depicted in red, green, cyan, and purple, respectively. (D) Loading plot for PC1 and PC2 components. This plot provides information about the contribution of each variable (Raman peak) to the principal components to identify the key features that differentiate the samples. The loading plot can be correlated to the changes observed in the Raman spectra, providing a comprehensive understanding of the molecular changes and interactions.

The study used Raman spectroscopy to investigate the binding of GMO-coated MENG (GMO@MENG) with DOX under the influence of a DC magnetic field, as schematically represented in Figure 3A. The samples were prepared, exposed to the magnetic field, and analyzed using Raman spectroscopy with the process mentioned in Section 2.5. The Raman spectra for GMO@MENG, DOX, and GMO@MENG with DOX are presented in Figure 3B in the presence and absence of a magnetic field. Remarkably, the Raman spectra of GMO@MENG and DOX with GMO@MENG in the absence and presence of a magnetic field exhibited spectral changes indicative of the differential interaction of drug molecules with the nanoparticles. Specifically, the changes observed in the Raman spectra of DOX at 437, 1089, 1202, and 1431 cm^–1^ suggest the binding of the GMO@MENG with DOX in the presence of a magnetic field. Other bands of DOX below 500 cm^–1^ are attributed to C–O and C=O bonds, which overlap considerably with the GMO@MENG spectrum. Skeletal ring vibrations of DOX are also observed between 1400 and 1500 cm^–1^. Modes between 1050 and 1300 cm^–1^, corresponding to C–O, C–O–H, and C–H, provided insights into the interaction of DOX with the nanoparticle surface.^59^ Changes in the typical Raman spectra region are highlighted in Figure 3B. Peaks around 1080–1100 and 1635 cm^–1^ attributed to the C–N stretching vibration, bending vibration of C–C=O, C–C, and the ring breathing vibration mode of the DOX molecule, showed variations in the presence of the magnetic field (see the yellow shaded portion in Figure 3B). The peaks between 1400 and 1500 cm^–1^ also showed changes due to the aromatic hydrocarbons’ C=C and C–C stretching vibration on interacting with the GMO@MENG in the presence of a magnetic field. The peak at 437 cm^–1^ is related to the vibration of the phenyl ring, C–C–O, and C–O bond, as highlighted in the blue region.^60,61^

The principal component analysis (PCA) plots derived from Raman spectra for all of the samples are depicted in Figure 3C. The PC1 component distinctly separates DOX from all other components. Clusters of GMO@MENG and GMO@MENG with DOX, both in the presence and absence of a magnetic field, are differentiated by the PC2 component. In the case of GMO@MENG bound with DOX in the presence of a magnetic field, the PC2 component leans slightly toward the higher positive side, indicating that positive peaks in the PC2 loading change the most when GMO@MENG binds with DOX. The peaks labeled in green in Figure 3D were mostly perturbed due to the interaction of GMO@MENG with DOX in the presence of the magnetic field. The positive peaks at 437, 1089, and 1202 cm^–1^ in the PC2 loading plot underwent significant changes upon interacting with the DOX. This observation may be attributed to the interaction of DOX’s oxygen atoms with the hydroxyl or carbonyl functional group of the glycerol monooleate molecules coated on the MENG nanoparticles. Moreover, it is also due to the higher concentration of the DOX on the nanoparticle’s surface in the presence of a magnetic field due to ionic interactions, as discussed earlier. Collectively, the Raman and PCA analyses confirm the changes in the mode of interaction between GMO@MENG and DOX in the presence and absence of a magnetic field, corroborating the magnetic field-induced loading and unloading of the drug molecules on the nanoparticle surface. This comprehensive analysis provides valuable insights into the molecular-level changes and interactions occurring during the binding of GMO@MENG with DOX under magnetic field conditions. Further, using Fourier transform infrared spectroscopy (FTIR) analysis of GMO@MENG, MENG, and GMO@MENG with DOX binding under the influence of a DC magnetic field are presented in the Supporting Information of Figure S6. The peak shifts are in good agreement with the observations made by FTIR and Raman spectroscopy.

### 3.3 FESEM-EDX Analysis for Drug Loading and Release Kinetics

**Figure 4:**
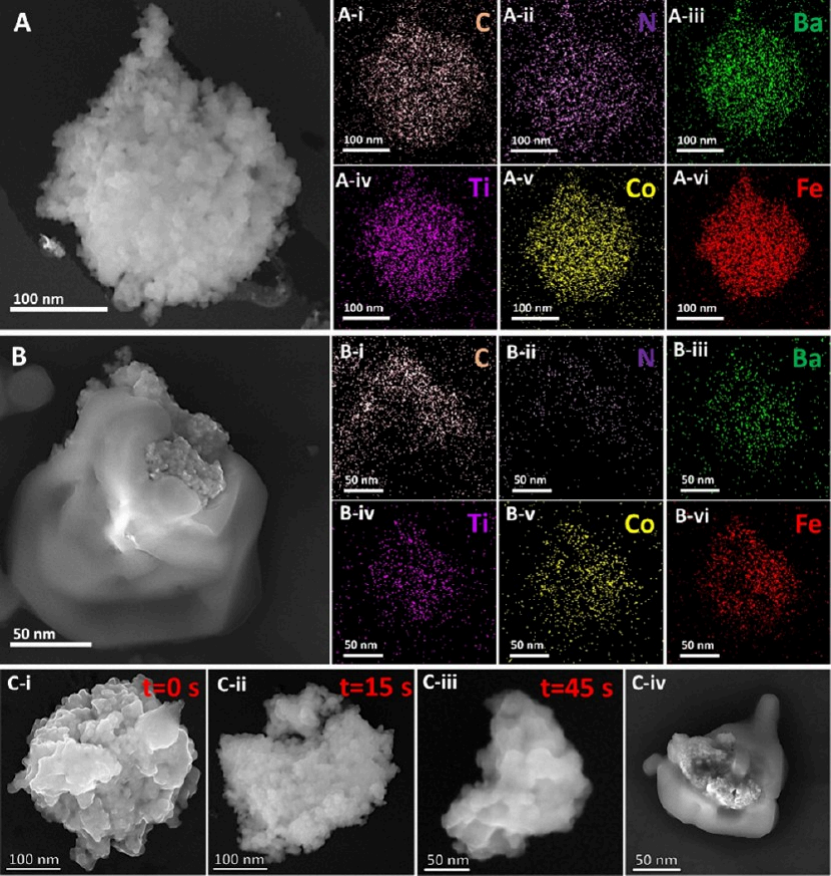
(A) FESEM-EDX illustration of drug conjugation in the MENG. (A(i–vi)) Elemental mapping of drug-coated MENG using SEM-EDX analysis, displaying varying elemental compositions such as C, N, Ba, Ti, Co, and Fe. (B) FESEM image of MENG after drug release under −100 Oe magnetic fields. (B (i–vi)) Elemental mapping of MENG after drug release under unidirectional application of −100 Oe magnetic fields using SEM-EDX analysis, revealing distinct elemental compositions. (C) FESEM image of time-dependent drug release under applied field. (C-i) At *t* = 0 s, drug-coated MENG shows uniform drug coating on the surface under rotating magnetic field (*H*_DC_ = 100 Oe) excitation. (C-ii) Partial drug detachment observed under a unidirectional magnetic field (*H*_DC_ = −100 Oe) excitation for 15 s. (C-iii) High-quantity drug release from the surface of DOX-coated MENG when exposed to the unidirectional magnetic field (*H*_DC_ = −100 Oe) for 45 s. (C-iv) Significant percentage of drug release was observed under a rotating magnetic field (*H*_DC_ = −100 Oe) application, with minimal residual traces of the drug.

FESEM microstructure analysis and FESEM-EDX analysis (Figure 4) depict the drug-loading and release kinetics of MENGs influenced by magnetic field exposure. Samples for FESEM measurements were prepared as mentioned in Section 2.8. Figure 4A presents the DOX-GMO@MENG-H under *H*_DC_ = 100 Oe applied magnetic field, illustrating the surface morphology of DOX uniformly coated on GMO@MENG. Elemental mapping in FESEM, as shown in Figure 4A (i–ii), verifies the uniform coating of DOX on the surface of GMO@MENG with the elemental distribution showing the presence of C and N, which are the primary constituents of the DOX nanocomplex. The presence of Ba, Ti, Co, and Fe elements in FESEM-EDX measurements constitutes part of the MENG, as depicted in Figure 4A (iii–vi). Further, the drug release mechanisms of MENGs are governed by an ionic bond-breaking mechanism by responding to the external magnetic field (*H*_DC_ = −100 Oe). Batch 4 was prepared with DOX-GMO@MENG-H and exposed to the rotational magnetic field (*H*_DC_ = −100 Oe) and drop-cast on a Si wafer, a process mentioned in Section 2.8. As depicted in Figure 4B, the FESEM-EDX analysis of batch 4 sample shows the uniform release of DOX from the surface of GMO@MENG in which DOX-GMO@MENG-H was exposed to the rotational magnetic field. This results in the modulation of the electric dipole in the form of polarity switching of the electric dipole from positive to negative on either side of the surface of the MENG. This dipole polarity switching disrupts the original charge symmetry within the MENG surface, leading to the breakage of ionic bonds. FESEM-EDX analysis in Figure 4B (i–ii)) confirms that the percentage composition of the primary constituents (C and N) of the DOX diminishes significantly, and only a little remaining drug is left on the tip of the GMO@MENG, indicating the release of drug from DOX-GMO@MENG-H composition. Figure 4B (iii–vi) shows the presence of the primary elements Ba, Ti, Co, and Fe of MENG.

Additionally, time-dependent drug release studies under unidirectional and rotating magnetic field excitation were conducted using batches 2, 3, and 4, as shown in Figure 4C (i–iv). Figure 4C (i) represents the uniform DOX drug coating of batch 1 at *t* = 0 s, i.e., DOX-GMO@MENG-H, signifying a uniform drug coating. In Batch 2, under the application of a unidirectional magnetic field, i.e., *H*_DC_ = −100 Oe, with reverse polarity as compared with the magnetic field exposed while loading the drug, the directionality of polarity of dipoles reverses across the surface of MENGs. This leads to the weakening of the ionic bond between the MENGs and the drug nano complex and results in the directional release of the drug from the surface of the MENG. The percentage of drug release progressively increases in the same direction with prolonged exposure under the applied magnetic field strength as observed in batch 3 samples. FESEM images in Figure 4C (ii) show initiation of drug release in the direction of applied magnetic field (*H*_DC_ = −100 Oe) exposed for at *t* = 15 s (batch 2) whereas increased drug release can be observed from the surface of GMO@MENG in the same direction in Figure 4C (iii) when exposed for longer time (*t* = 45 s) to the magnetic field (*H*_DC_ = −100 Oe), i.e., batch 3 samples. Furthermore, due to the exposure to rotational magnetic field (*H*_DC_ = −100 Oe) for 2 min in batch 4 samples, it was observed that all ionic bond symmetry is completely disrupted by reducing the binding energy and reorienting all ionic bonds with the DC magnetic field, significantly enhancing drug release efficiency throughout the surface of MENG as shown in Figure 4C (iv).

### 3.4 AFM Analysis of Drug Release Using a Unidirectional and Rotating Magnetic Field

**Figure 5:**
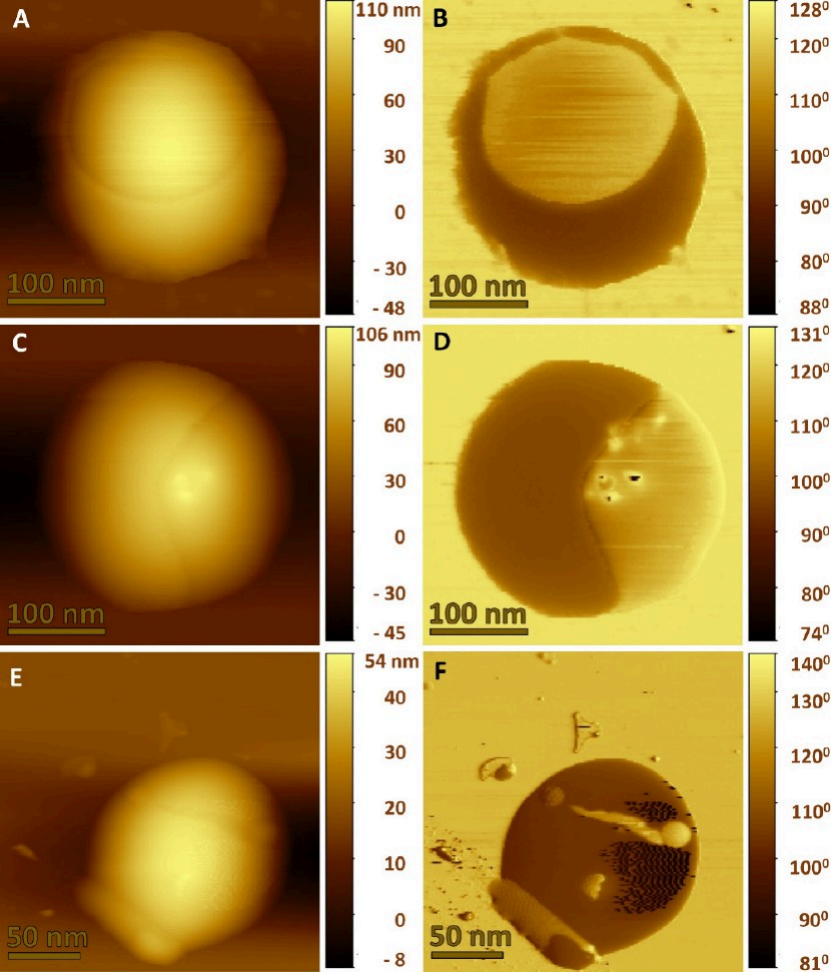
(A) Topographical image showing directional drug release from the surface of MENG-GMO–DOX when exposed to unidirectional magnetic field *H*_DC_ = −100 Oe for 15 s. (B) Phase image reveals a clear differentiation between phases of MENG and surface-coated DOX. (C) Topographic image depicting orthogonal drug release achieved through the application of a unidirectional magnetic field set at −100 Oe. (D) Phase image illustrating the mechanism of drug release from GMO-MENG in an orthogonal fashion. (E) Topographic image demonstrating a remarkable 98% drug release under the influence of a rotating magnetic field. (F) Phase image indicating the drug release with only negligible DOX traces remaining.

The AFM measurement (Figure 5) provides confirmational evidence of the effect of a unidirectional and rotating magnetic field on the controlled drug release from the surface of DOX-GMO@MENG-H. As discussed in Section 2.9, batch 1 was exposed to a unidirectional magnetic field (*H*_DC_ = −100 Oe) for *t* = 45s, drop-cast over silicon wafer, and analyzed using AFM. From Figure 5A, the AFM topography signifies the unidirectional DOX drug release from the surface of DOX-GMO@MENG-H due to ionic bond breakage. The AFM topography image clearly signifies the creation of a void area at the top surface of the DOX-GMO@MENG-H resulting in height differences. However, the AFM phase image (Figure 5B)) clearly signifies the phase difference between the DOX-coated and DOX-released section of the analyzed single DOX-GMO@MENG-H and that the DOX molecule is detached from the DOX-GMO@MENG-H in the applied magnetic field direction. In Figure 5C, another DOX-GMO@MENG-H from the same batch 1 was also analyzed, which was automatically positioned orthogonally as compared to DOX-GMO@MENG-H in Figure 5A. In Figure 5C,D, there is evidence of the drug release in the direction of the applied unidirectional magnetic field. Interestingly, in the AFM phase image (Figure 5D), a few of the drug molecules are still left on the DOX unloaded section of the DOX-GMO@MENG-H. Furthermore, due to the rotational magnetic field applied (*H*_DC_ = −100 Oe) for a duration time of 2 min in batch 2 samples, all ionic bonds between DOX molecules and GMO@MENG are broken, and most of the DOX drug has been released from the surface of DOX-GMO@MENG-H, leaving behind the small observable traces of DOX molecules. This observation clearly strengthens the experimental findings from spectrophotometry and FESEM measurements. Such a unique and detailed analysis of magnetically controlled nanoscale drug release from a nanocarrier is seldom reported in the literature, and additionally, the AFM analysis verifies the magnetic field direction-modulated directional and controlled drug release from the surface of a single MENG nanoscale, suggesting the potential applicability of the drug release technique at the subcellular level.

### 3.5 Percentage BSA Absorption and In Vitro Cytocompatibility Studies

**Figure 6:**
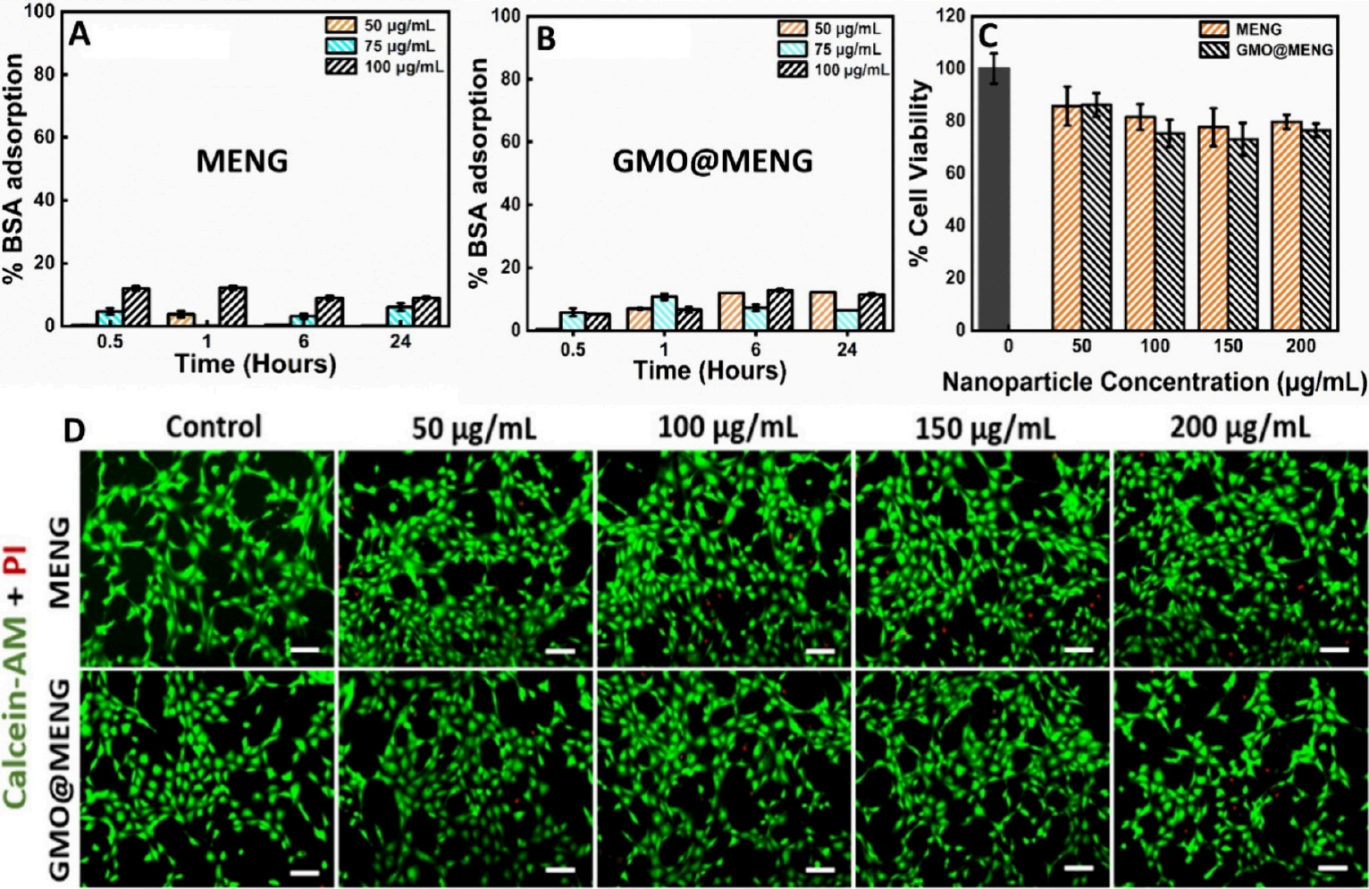
Protein adsorption assay utilizing BSA was conducted over 24 h, assessing various concentrations of (A) MENG and (B) GMO@MENG. Cytocompatibility studies including (C) MTT assay and (D) live–dead assay were performed across different concentrations (ranging from 0 to 200 μg/mL) of MENG and GMO@MENG in the NIH-3T3 cell line. The scale bar is 100 μm. The studies were performed in triplicates, and data have been presented as the mean ± standard deviation.

BSA was employed to assess the adsorption mechanisms of MENG since albumin is abundantly present in the human serum. Protein adsorption on the nanoparticle surface was evaluated by incubating the nanoparticles in a BSA solution for 24 h. The process is mentioned in detail in Section 2.13. The findings revealed that both MENGs and GMO@MENG exhibited minimal adsorption, ranging from 10 to 11% of BSA on their surfaces, and this level remained relatively constant over time (Figure 6A,B). This low protein adsorption is significant for drug carriers activated by external stimuli.

In vitro cytocompatibility was evaluated by employing 3-(4,5-dimethylthiazol-2-yl)-2,5-diphenyltetrazolium bromide (MTT) and live–dead assays on murine fibroblast (NIH-3T3) with a process described in Section 2.12. Cells were incubated with MENGs and GMO@MENG for 24 h. The MTT assay results, illustrated in the bar graph, showcased a remarkable cell viability of over 75% across all concentrations tested for both MENGs and GMO@MENG, as shown in Figure 6C. Furthermore, fluorescence microscopy images in Figure 6D corresponding to Calcein-AM (green dye) and PI (red dye) staining depicted a predominantly green-stained cell and a negligible number of cells-stained red. This strongly indicates that most cells were healthy and viable in the presence of various concentrations (50, 100, 150, and 200 μg/mL) of MENG. Both cell viability assays collectively affirm the cytocompatibility of these MENGs at different concentrations. Since these MENGs are intended for in vitro studies with a potential application in biological environments, it becomes crucial to assess their interaction with proteins. Additionally, the proteins present in biological fluids play a role in the in vitro biodistribution of MENGs. The colloidal stability of GMO@MENG was investigated in DPBS with a pH of 7.4 and a 10% FBS solution using dynamic light scattering measurements using the Malvern Nano ZS Zetasizer. Particle size distribution was measured at 24 h intervals using DLS. As depicted in the Supporting Information of Figure S7, the hydrodynamic size of the MENGs showed no significant change over 24 h, indicating their stability in both media.

### 3.6 In Vitro Anticancer Activity of DOX-GMO@MENG-H

**Figure 7:**
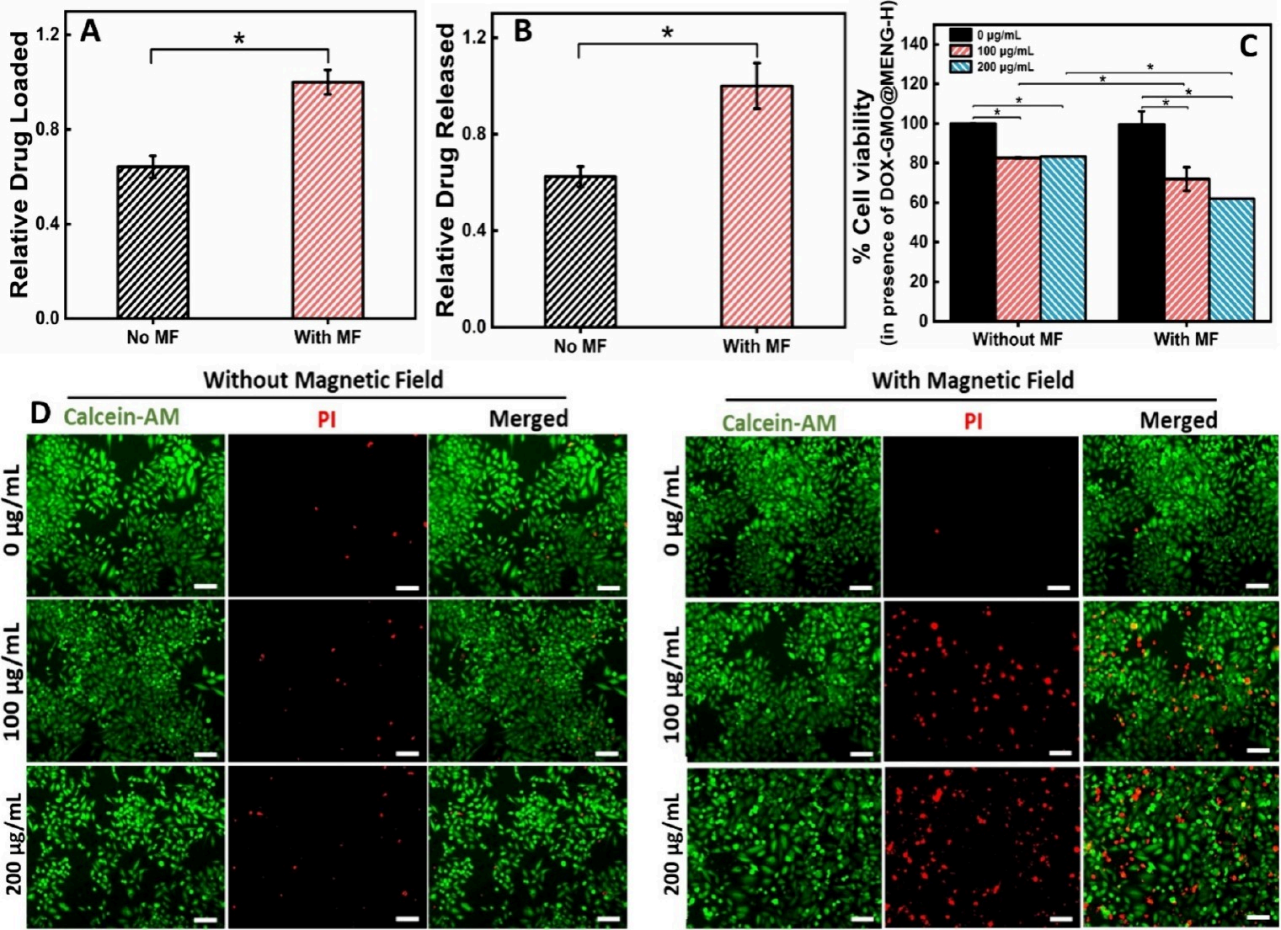
Relative amount of (A) DOX loaded onto GMO@MENG and (B) DOX released from DOX-GMO@MENG-H were assessed both in the presence and absence of an external magnetic field (*H*_DC_ = −100 Oe). The anticancer activity of DOX-GMO@MENG-H in the breast cancer cell line (MCF-7) was examined through (C) MTT assay and (D) live–dead assays under conditions under the application of the rotating magnetic field (*H*_DC_ = −100 Oe) for 2 min. The scale bar is 100 μm. (*p < 0.05).

Following the preparation of GMO@MENG, DOX was loaded onto the GMO@MENG in the presence of a magnetic field (*H*_DC_ = 100 Oe) and applied externally in a clockwise rotational direction for 5 min. The process is mentioned in detail in Section 2.14. The drug-loaded percentage was calculated, and relative drug loading was plotted for DOX-GMO@MENG and DOX-GMO@MENG-H. Remarkably, it was observed that the drug loading under magnetic field stimulation was 1.55 times more than that without any stimuli, as illustrated in Figure 7A. Furthermore, the amount of DOX released from DOX-GMO@MENG-H under a magnetic field stimulus of *H*_DC_ = −100 Oe was assessed by recording the absorbance of the supernatant containing the released drugs from these MENGs. A 2 min magnetic field (*H*_DC_ = −100 Oe) stimulation resulted in 1.6 times higher drug release than conditions without stimuli, as depicted in Figure 7B.

To evaluate the enhanced DOX drug release in vitro and to analyze its effect on cancer cells, the breast cancer cell line (MCF-7) was exposed to DOX-GMO@MENG-H and subjected to a rotational DC magnetic field (*H*_DC_ = −100 Oe) for 2 min. Following 24 h of incubation with these magnetically stimulated MENGs, cell viability was evaluated via MTT assay and live–dead staining to observe the anticancer effect of released DOX. More than 80% cell viability was observed for cells treated with DOX-GMO@MENG-H without magnetic stimulation for both concentrations (Figure 7C). However, a significant decrease in cell viability, ranging from 60 to 65%, was noted for cells treated with DOX-GMO@MENG-H and exposed to reverse polarity magnetic field (*H*_DC_ = −100 Oe) stimulation. The percentage of cell viability under these conditions was found to be equivalent to untreated cells not exposed to the magnetic field. To determine the impact of the external magnetic field alone (of comparable intensity and duration), untreated cells were exposed to the magnetic field, revealing a negligible effect on the cell viability. Fluorescence microscopic images presented in Figure 7D further supported these findings. Cells treated with DOX-GMO@MENG-H under rotational DC magnetic field stimulation of the strength −100 Oe exhibited a higher number of dead cells, evidenced by the red staining from propidium iodide (PI), indicating the anticancer effect of enhanced DOX released from the nanogenerators. These studies underline that, under magnetic field exposure, a higher amount of anticancer drug is released, leading to increased cancer cell killing compared with conditions without magnetic field exposure. However, based on cell viability against different drug exposures, the IC-50 value was found to be 250 μg/mL. The data are well described by a polynomial equation with an exponential decay term, showcasing a strong fit with an *R*-squared value of 0.98. (Supporting Information in Figure S8). Consequently, this magnetic-field-stimulated drug carrier (DOX-GMO@MENG-H) holds promising results for on-demand and controlled drug delivery in anticancer therapy.

## 4 Conclusions

In summary, our research has proposed an innovative technique for controlled and swift drug delivery utilizing the capabilities of MENGs in conjunction with a low-intensity direct current magnetic field. The core@shell nanostructured MENGs serve as field-responsive nanocarriers for doxorubicin drug release. The key to their functionality lies in the unique magnetoelectric properties that allow these nanogenerators to generate an electric dipole on their surfaces when exposed to a magnetic field. This distinctive feature enables the directional modulation of the surface electrical dipole on the surface of MENG excited by the magnetic field to achieve on-demand drug release by facilitating the creation or breaking of ionic bonds. Also, their strong conjugation with the drug molecule through ionic bonding, activated by a low-intensity remote magnetic field, is an unprecedented feature. This capability has unprecedented advantages for achieving the release of drug molecules with exceptional precision in subtle and complex regions while effectively preventing premature drug release. Furthermore, the utilization of a low-(DC) magnetic field in the technique simplifies the process, making it more applicable in the medical field, by avoiding the methods that involve exposing cells to high-intensity alternating current (AC) magnetic fields using large Helmholtz coils and complex setup. To visualize and analyze the magnetization, electrification, and nanoscale magnetoelectric properties of MENGs, state-of-the-art in situ off-axis electron holography under Lorentz field conditions was conducted. Such unique microscopic techniques to analyze nanoscopic electromagnetic properties have seldom been reported in the literature. The magnetic field-assisted drug-loading process was extensively analyzed by Raman spectroscopy, and the positive peaks identified in PCA analysis affirmed the molecular-level interactions of GMO@MENG with DOX, establishing ionic bonds under the influence of a directional magnetic field.

Further, time-dependent drug release studies using FESEM-EDX and AFM techniques under unidirectional and rotational magnetic field excitation provide great insight into the controlled release of DOX from the MENG surface over specific time intervals of directional magnetic field exposure. Notably, under a rotational magnetic field (−100 Oe) for 2 min, the maximum release of DOX was confirmed and provides valuable confirmations of the responsiveness and effectiveness of the developed drug delivery system. Additionally, drug release kinetics were monitored through spectrophotometry, where the difference in absorbance at 480 nm was recorded in the presence and absence of a magnetic field. These methods provided a comprehensive understanding of the electrodynamics involved in drug attachment and detachment from the nanocarriers under the influence of a magnetic field. The investigation on percentage protein adsorption on the MENG’s surface using BSA, and the findings revealed a minimal protein adsorption rate on the surface of MENGs, thereby preventing their engulfment by macrophages. Cytocompatibility testing of the MENG conducted with NIH-3T3 cell lines using the MTT assay and live–dead testing shows cell viability exceeding 75%, thus strongly suggesting the biocompatibility of MENGs. In vitro studies have demonstrated that magnetically assisted on-demand doxorubicin drug delivery near MCF-7 breast cancer cells significantly enhanced cancer cell killing efficiency under rotating magnetic field excitation. This promising outcome underscores the potential of our method to revolutionize controlled drug delivery strategies in the realm of cancer therapy. In essence, our research not only contributes to the fundamental understanding of magnetoelectric nanogenerators but also lays the foundation for a groundbreaking approach to precision drug delivery in the context of cancer treatment. The experimental outcomes presented in this research will contribute to the fundamental understanding of magnetoelectric nanogenerators, as well as laying the foundation for a groundbreaking approach to high precision, targeted, and swift drug delivery in the context of cancer treatment.

## Supporting Information Available

Off-axis electron holography measurements, DLS and zeta potential measurements, AFM measurements, XRD measurements, VSM measurements, FTIR measurements, particle stability in DPBS and FBS, and IC50 value calculation (PDF)

## Abbreviations

MENG: magnetoelectric nanogenerator
DOX: doxorubicin
GMO: glyceryl monooleate
FBS: fetal bovine serum
MTT: 3-(4,5-dimethylthiazol-2-yl)-2,5-diphenyltetrazolium bromide
DOX-GMO@MENG-H: magnetically exposed GMO@MENG sample
FESEM-EDS: field-emission scanning electron microscope energy-dispersive X-ray spectroscopy
AFM: atomic force microscopy
HRTEM: high-resolution transmission electron microscopy
VSM: vibrating sample magnetometer
OEH: off-axis electron holography
STEM: scanning transmission electron microscopy
CFO: CoFe_2_O_4_
BTO: BaTiO_3_
PCA: principal component analysis
BSA: bovine serum albumin
H_DC_: direct current magnetic field
