# Supplementary material for: Remotely Controlled Surface Charge Modulation of Magnetoelectric Nanogenerators for Swift and Efficient Drug Delivery
Source: ACS Omega. 2024 Jun 15;9(26):28937–50. doi: 10.1021/acsomega.4c03825 (PMC11223158; doi:10.1021/acsomega.4c03825)
Supplement: Supplementary file 1 — ao4c03825_si_001.pdf [file ao4c03825_si_001.pdf]

Supporting Information

Remotely controlled surface charge modulation of magnetoelectric nanogenerator for swift and efficient drug delivery

Nandan Murali[a], Simran Kaur Rainu[b], Arti Sharma[c], Soumik Siddhanta[c], Neetu Singh[b] and Soutik Betal[a*]

[a] Department of Electrical Engineering, Indian Institute of Technology Delhi, Hauz Khas, New Delhi-110016

[b] Center for Biomedical Engineering, Indian Institute of Technology Delhi, Hauz Khas, New Delhi-110016

[c] Department of Chemistry, Indian Institute of Technology Delhi, Hauz Khas, New Delhi-110016

*E-mail: soutik@ee.iitd.ac.in

The supporting file includes:

- Supplementary Note 1: Off-axis Electron Holography measurements
- Supplementary Note 2: DLS and Zeta potential measurements
- Supplementary Note 2: AFM measurements
- Supplementary Note 4: XRD measurements
- Supplementary Note 5: VSM measurements
- Supplementary Note 6: FTIR measurements
- Supplementary Note 7: Particle stability in PBS and FBS
- Supplementary Note 8: IC50 value

Supplementary Note 1: Off-axis Electron Holography measurements

Off-axis electron holography is performed using scanning transmission electron microscopy (STEM) with a probe-corrected microscope (JEOL-ARM200F). The STEM was equipped with a biprism (platinum wire) connected with a voltage source, a Lorentz lens attached to the microscope, and Gatan Digital micrograph software with a beta version of HoloWorks 5.0.7. When referring to magnetic materials, electron holography is required to be performed under Lorentz mode (field-free condition), this means with the objective lens turned off to keep undisturbed the remnant magnetization state of the sample and using the Lorentz lens with the magnetic field of 1.5 Tesla. The residual magnetic field of the objective lens was measured to be approximately 50 Oe. The holograms must be acquired using high fringe contrast (34%) – Supplementary Figure 1 A, which can be adjusted with a bias voltage applied to the biprism. Specifically for MENG, a biprism voltage of 40 V was used to cover the object's field of view. The Fresnel fringes created have a width of 140 nm. In our experiment, a single MENG has been oriented longitudinally in a

parallel direction of the interference fringes as shown in Supplementary Figure 1 B. To enhance the phase resolution, single exposures with exposure times ranging from 2 to 4 seconds were used for registered hologram acquisition. The holograms were recorded with Gatan's software Digital Micrograph and reconstructed by a beta version of HoloWorks 5.0.7, featuring a function for extracting magnetization and electrostatic potential from phase images at several frames per second. Every retrieved phase was numerically reconstructed using a reference hologram and the object hologram to remove the influence of the perturbed reference wave as shown in Supplementary Figure 1C. The phase separation between the electrostatic potential and the magnetic field is achieved by manually flipping (up and down) the sample, i.e., the TEM grid containing the MENG. The phase extracted from the up or down flip state of the sample contains both magnetic and electrostatic phases. The magnetization was extracted by subtracting the phase maps obtained from the two flip states (up and down). The magnetization direction was mapped and presented with a color map indicating direction (Supplementary Figure 1D) The electrostatic potential generated by the MENG was extracted by adding the phase maps obtained from the two flip states (up and down).

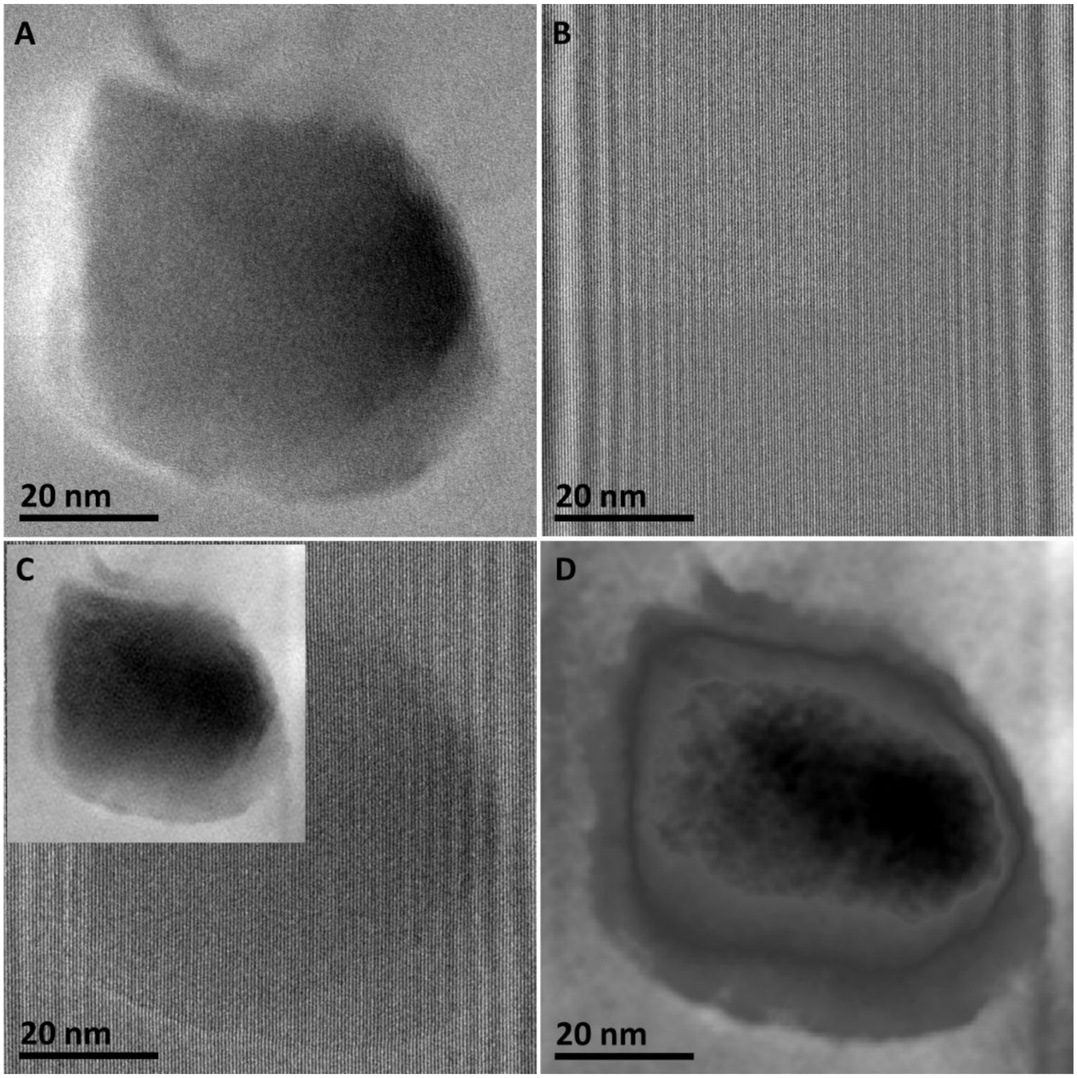

Figure S1: Off-axis electron holography under Lorentz field conditions (A) STEM image of a single MENG **(B)** The high fringe contrast (34%) and width (140 nm) adjusted with a bias voltage applied to the biprism. (C) Extracted electromagnetic phase (inset) from the hologram of MENG phase in Fresnel fringes. (D) Electrical property phase extracted from the addition of the flipped and un-flipped electromagnetic phases of a single MENG.

Supplementary Note 2: DLS and Zeta potential measurements

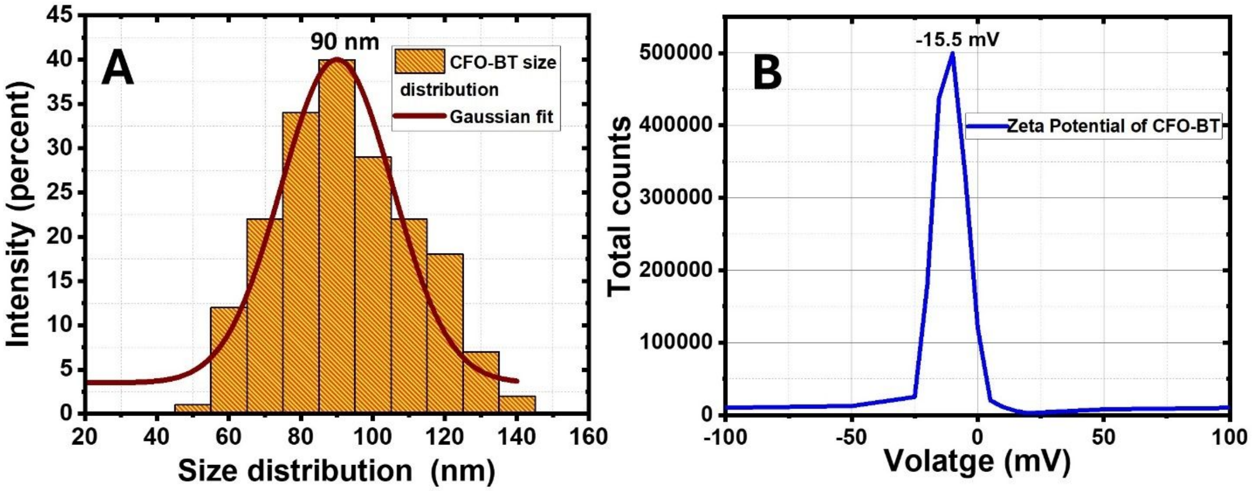

Figure S2: Zeta measurements MENGs. (A) size distribution. (B) Zeta potential

Figure S2 shows Particle size distribution and zeta potential measurements were conducted using a Malvern Instruments Zetasizer Nano ZS. The zeta potential value reflects the extent of electrostatic repulsion between neighboring particles with similar charges in a dispersion.[4] The nanoparticle samples were diluted with ethanol and exhibited a size distribution of 90 nm (Figure A). Figure B presents zeta potential and was determined using the Dynamic Light Scattering (DLS) mode, and zeta potential is found to be around -15.5 mV.

Supplementary Note 3: AFM measurements

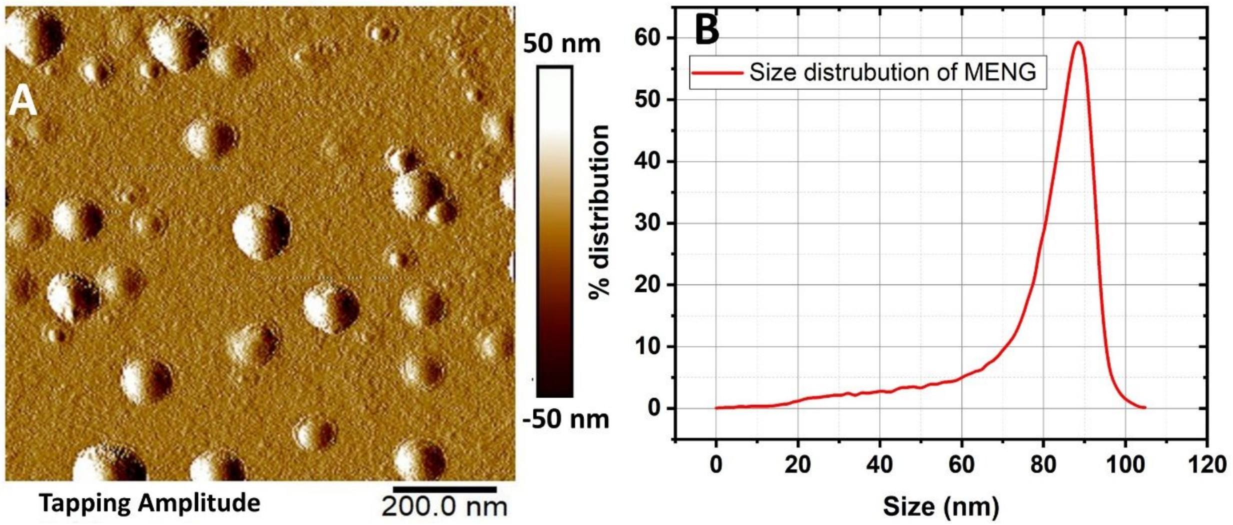

Figure S3: (A) AFM tapping amplitude image of MENG. (B) Size distribution of MENG

MENGs were dispersed in ethanol and sonicated for 30 mins. Si wafer measuring 1 cm x 1 cm, was used, and an ethanolic mixture was drop cast forming a thin film for AFM measurements. The AFM analysis of the MENG surface was conducted in noncontact mode and the tapping amplitude image is depicted in Figure Supplementary Figure 3 (A), and it revealed smooth surfaces of MENG with a surface roughness of less than 1 nm. The presence of large and small spherical structures indicated the formation of a core-shell structure. The depth histograms illustrated the density distribution of data points on the surface, and the average size distribution was determined to be 90 nm, as illustrated in Supplementary Figure 3 (B).

Supplementary Note 4: XRD measurements

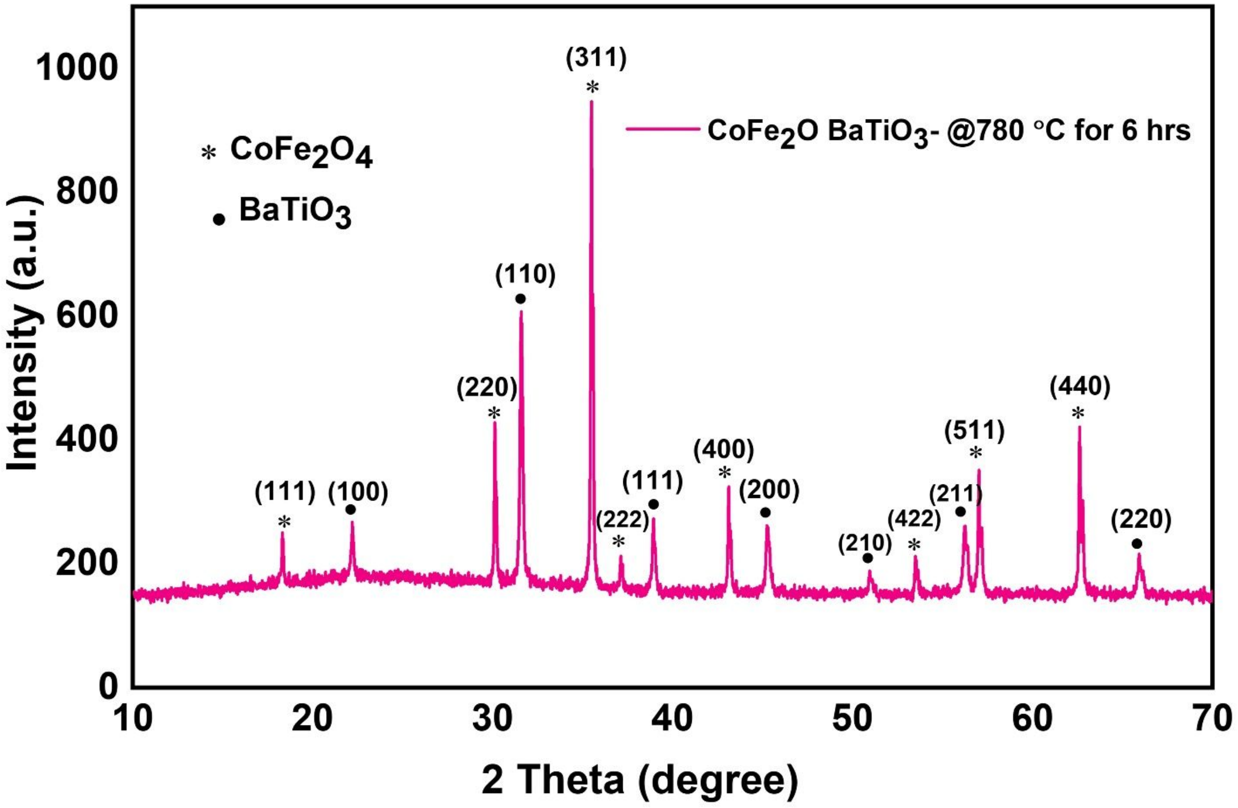

Figure S4: X-ray diffraction patterns for MENGs indicating the planes of $CoFe_2O_4$ (*) and $BaTiO_3$ (□) pattern.

The crystalline phase formation of MENGs was assessed through X-ray diffraction (XRD), as illustrated in Supplementary Figure 4. The observed XRD patterns were identified with two distinct reflections: the core $CoFe_2O_4$ phase corresponds to the $Fd\bar{3}m$ space group (COD ID:

1533163) and the ferroelectric shell $BaTiO_3$ phase associated with the *P4mm* space group (COD ID:1507757) having cubic and tetragonal crystal structures respectively. Notably, there was no indication of any impurity or intermediate phase in the analysis. The lattice parameters of the $BaTiO_3$ were found to be (a=b≠c) 4.018 Å and 3.809 Å., whereas the (c/a) ratio is also found as 1.05. Thus, according to the calculated lattice parameters and Bragg peaks.

Supplementary Note 5: VSM measurements

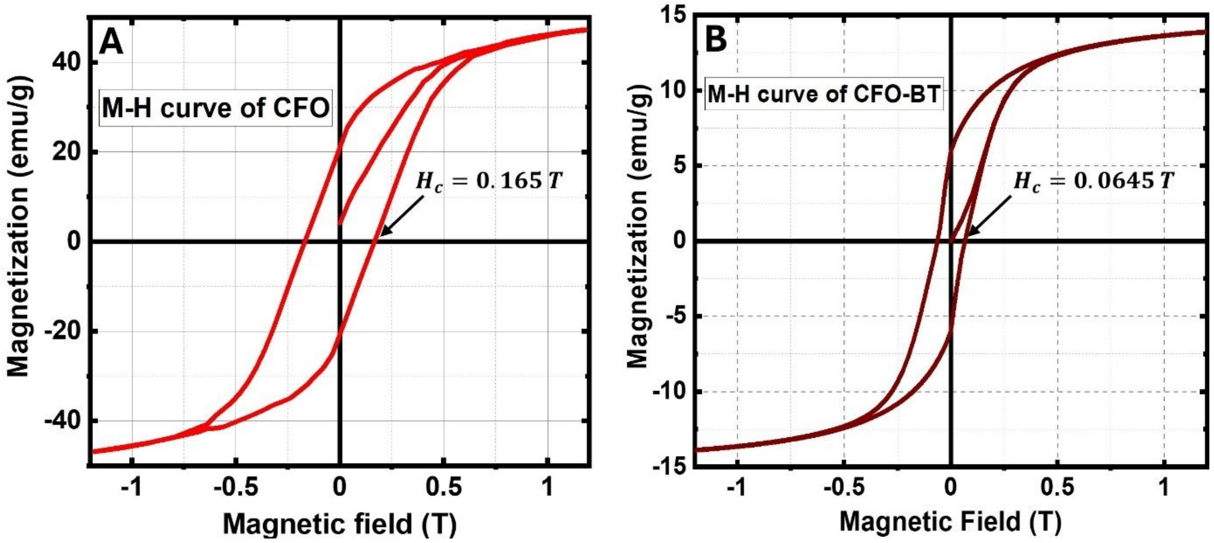

Figure S5. The magnetization vs. the magnetic field loop for (A) $CoFe_2O_4$ (B) $CoFe_2O_4$- $BaTiO_3$ core-shell MENPG under an applied magnetic field at room temperature, as measured by Vibrating Sample Magnetometer (VSM)

Magnetic hysteresis measurements were conducted to study the magnetic characteristics of MENGs, yielding a hysteresis curve depicted in Supplementary Figure S5. The observed hysteresis in Figure S5 (A) indicates non-linear ferromagnetic behavior, of CFO with remnant magnetization (Mr) and coercive field were determined to be 20.71 emu/g and 1756 Oe, respectively.

Additionally, in Figure S5 (B) the remnant magnetization (Mr) and coercive field of CFO-BT were determined to be 5.55 emu/g and 661 Oe, respectively. It is observed however that, the coercive field of CFO is higher than CFO-BT.[2]

Supplementary Note 6: FTIR measurements

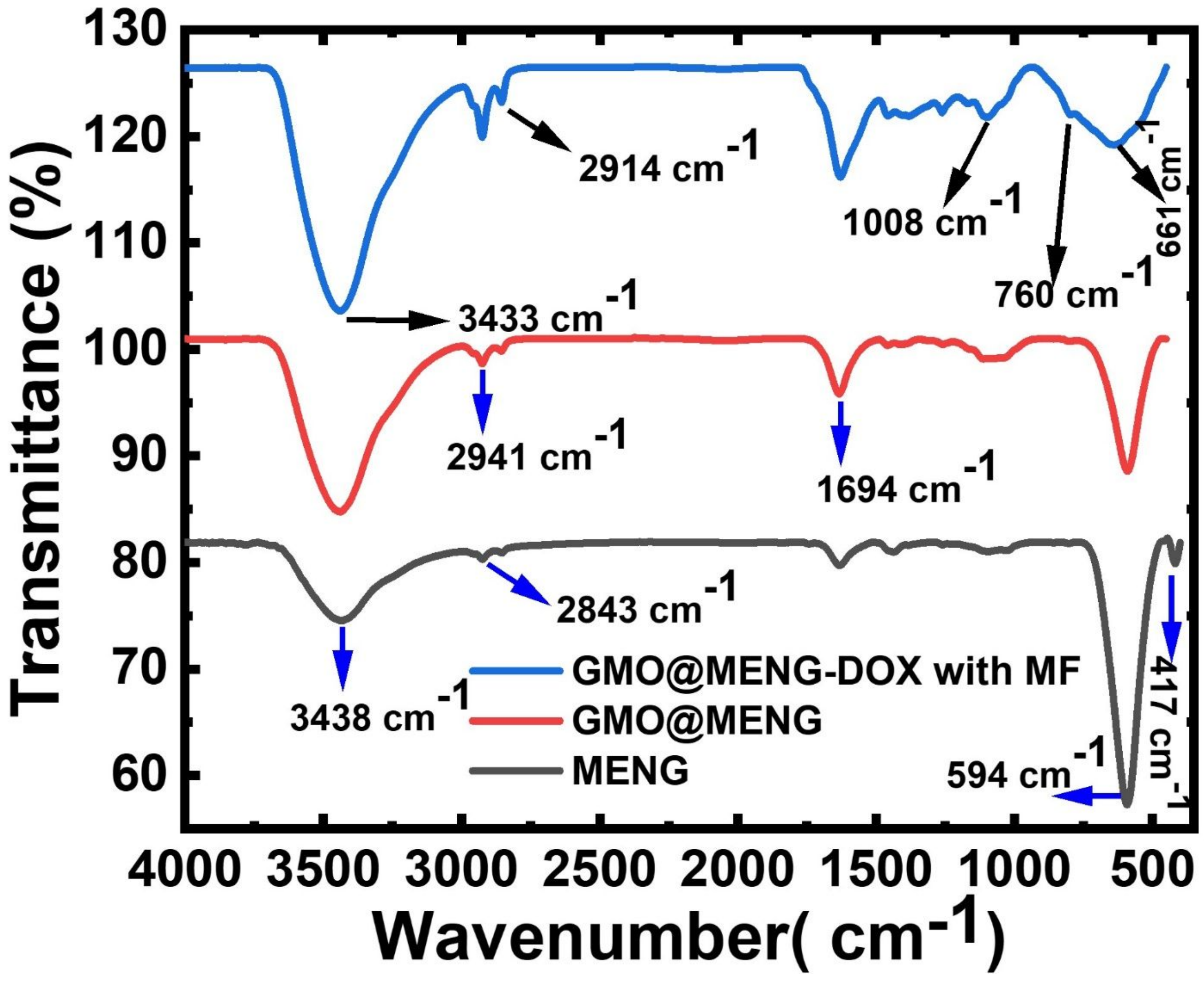

Figure S6. FTIR Spectra of MENG, GMO@MENG, and GMO@MENG-DOX

FTIR of MENG, GMO@MENG, and GMO@MENG-DOX under applied magnetic field has illustrated in The vibration of Fe-O was detected at 417 cm^{-1}, indicating tetrahedral sites, while the Co-O vibration band appeared at 594 cm^{-1}, suggesting octahedral sites. Additionally, Ba-O stretching and Ti-O bands were observed at 2849 cm^{-1} and 3438 cm^{-1}, respectively, confirming the complete reaction of $BaTiO_3$.[3]

The FTIR spectrum of the GMO@MENG surface exhibited peaks at 1694 and 2941 cm^{-1}, corresponding to the ester bond and CH_2 stretching modes, respectively, indicating the presence of the GMO layer on the MENG surface.[4] The FT-IR spectrum of DOX displayed multiple peaks at 3433, 2914, and 1008 cm^{-1}, corresponding to different quinone and ketone carbonyl groups. Distinguishing between the bands for quinine and ketone was challenging due to both having carbonyl groups. Peaks at 760 cm^{-1} and 878 cm^{-1} were attributed to amide I ($-NH_2$) wagging N−H deformation bonds, respectively. The 661 cm^{-1} peak corresponding to out-of-plane O-H bending confirmed the successful loading of DOX on the GMO@MENG under the application of a +100 Oe magnetic field.[5]

Supplementary Note 7: Particle stability in PBS and FBS

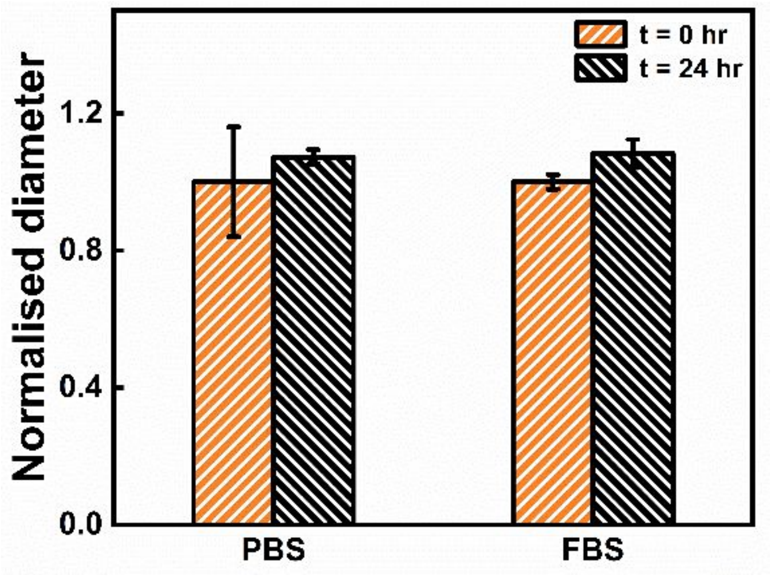

Figure S7. Stability of GMO@MENGs in PBS (pH 7.4) and 10 % FBS solutions at 24-hour time intervals. The stability of GMO@MENGs in PBS with a pH of 7.4 and 10% FBS solutions was assessed using the Malvern Nano ZS Zetasizer. Particle sizes of MENGs in each of these media were measured at both 0-hour and 24-hour time points.

Since these magnetic nanoparticles have to be utilized for in vitro studies and have ultimate application in biological environments, it is important to evaluate their interaction with the proteins. Moreover, the proteins present in the biological fluids also impact the biodistribution of the nanoparticles. Therefore, particle colloidal stability of GMO@MENG was studied in PBS 7.4 and 10 % FBS solution by measuring particle size distribution at 24-hour intervals using DLS. The figure showed that the hydrodynamic size of the nanoparticles did not change significantly over 24 hours, thus indicating its particle stability in both mediums.

Supplementary Note 8: IC50 value

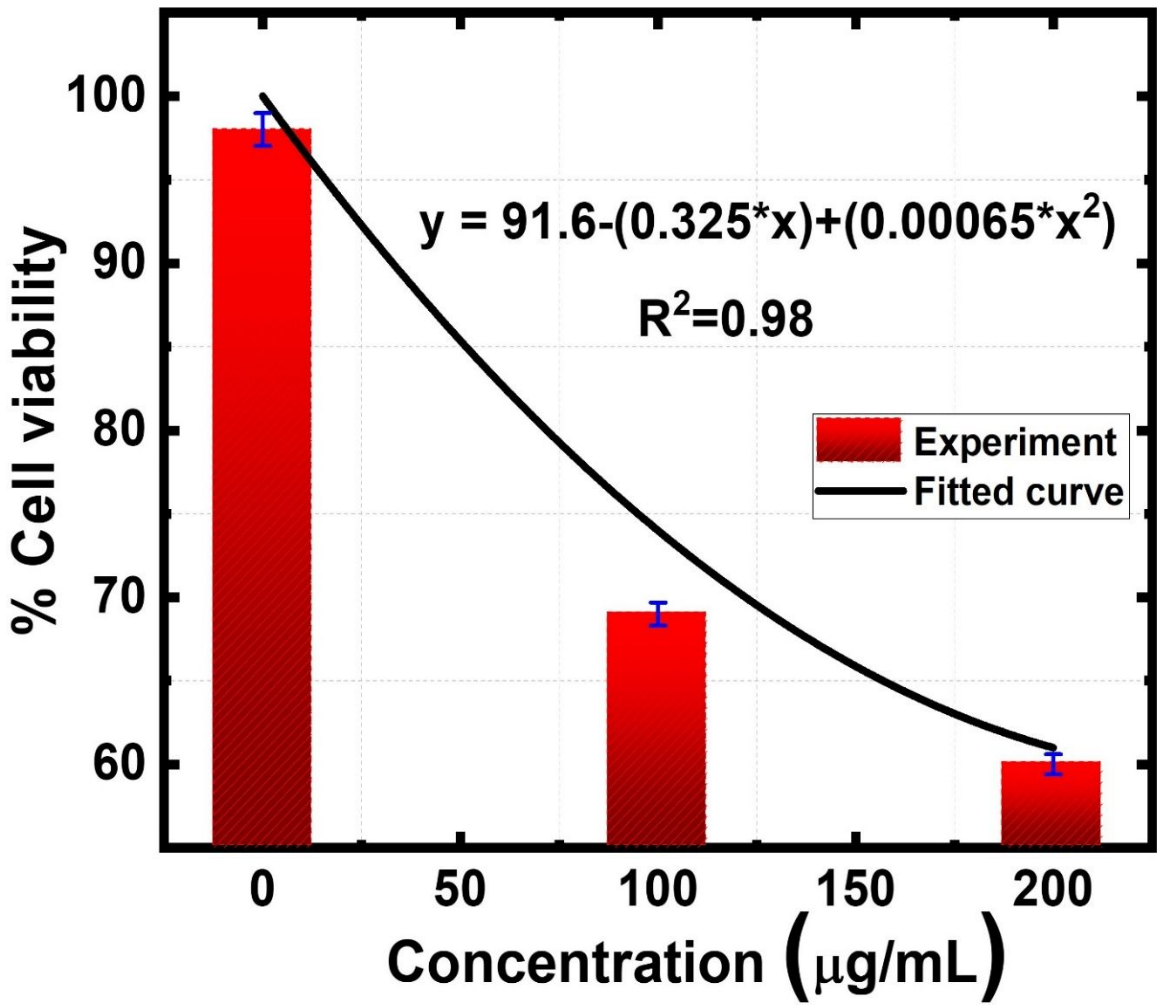

Figure S8. IC50 value calculation

As per the results, we have fitted the curve and with the help of the fitted equation as shown in Figure we found that the Ic-50 value is 250 μg/mL.

References

(1) Betal, S.; Shrestha, B.; Dutta, M.; Cotica, L. F.; Khachatryan, E.; Nash, K.; Tang, L.; Bhalla, A. S.; Guo, R. Magneto-Elasto-Electroporation (MEEP): In-Vitro Visualization and Numerical Characteristics. *Sci Rep* **2016**, *6* (1), 32019. DOI:10.1038/srep32019.

(2) Song, H.; Kim, D.; Abbasi, S. A.; Gharamaleki, N. L.; Kim, E.; Jin, C.; Kim, S.; Hwang, J.; Kim, J.-Y.; Chen, X.-Z.; Nelson, B. J.; Pané, S.; Choi, H. Multi-Target Cell Therapy Using a Magnetoelectric Microscale Biorobot for Targeted Delivery and Selective Differentiation of SH-SY5Y Cells via Magnetically Driven Cell Stamping. *Mater. Horiz.* **2022**, *9* (12), 3031–3038. DOI: 10.1039/D2MH00693F.

(3) Thotakura, R.; Rajendar, V.; Ramana murthy, S. CoFe2O4–BaTiO3 Multiferroic Composites: Role of Ferrite and Ferroelectric Phases on the Structural, Magneto Dielectric Properties. *Journal of Materials Science: Materials in Electronics* **2017**, *28*. DOI:10.1007/s10854-017-6983-6.

(4) Guduru, R.; Khizroev, S. Magnetic Field-Controlled Release of Paclitaxel Drug from Functionalized Magnetoelectric Nanoparticles. *Particle & Particle Systems Characterization* **2014**, *31* (5), 605–611. DOI:10.1002/ppsc.201300238.

(5) Songsurang, K.; Praphairaksit, N.; Siraleartmukul, K.; Muangsin, N. Electrospray Fabrication of Doxorubicin-Chitosan-Tripolyphosphate Nanoparticles for Delivery of Doxorubicin. *Arch. Pharm. Res.* **2011**, *34* (4), 583–592. DOI:10.1007/s12272-011-0408-5.
